# The Effect of Pubertal Status on Self‐Regulation of Behavior and Executive Functions—A Systematic Review

**DOI:** 10.1002/dev.70069

**Published:** 2025-08-12

**Authors:** Thiago F. A. França, Isis A. Segura, Natália M. Dias, Mônica C. Miranda, Sabine Pompeia

**Affiliations:** ^1^ Departamento de Psicobiologia Universidade Federal de São Paulo, Escola Paulista de Medicina São Paulo São Paulo Brazil; ^2^ Universidade Federal da Fronteira Sul, Campus Passo Fundo Passo Fundo Rio Grande do Sul Brazil; ^3^ Departamento de Psicologia Universidade Federal de Santa Catarina Florianópolis Santa Catarina Brazil; ^4^ Universidade Federal da Bahia Salvador Bahia Brazil

**Keywords:** adolescence, executive functions, puberty, self‐control, self‐regulation

## Abstract

Behavioral self‐regulation (SR) refers to a set of abilities that enable flexible, adaptive, and goal‐directed behavior, including the abilities known as hot (emotional regulation) and cool (e.g., controlled attention) executive functions (EFs). Such abilities mature during adolescence, a period marked by developmental brain changes due to learning/experience as individuals grow older, and by changes in sex hormone levels due to puberty, which influence brain maturation and can affect cognition. However, it is unclear to what extent the maturation of SR/EF is determined by adolescents’ stage of pubertal development—that is, their pubertal status—irrespective of their age. We investigate this issue through a systematic review of the literature. Searching PubMed, Web of Science, Scopus, and PsycINFO, we found 125 studies about the relationship between pubertal status and SR/EF. However, only 28 of these included results about pubertal status adjusted for the confounding effects of age. These studies were heterogeneous in their methods and reported mixed results with no clear patterns. The literature was also fraught with conceptual and methodological shortcomings. As a result, current evidence is inconclusive about pubertal status effects on SR/EF. We discuss the implications of these findings for current theories of adolescent cognitive development.

## 1 Introduction

Adolescence is an important developmental period that involves substantial changes in behavior accompanied by marked cognitive maturation (Crone 2009; Steinberg et al. 2018; Icenogle et al. 2019; Icenogle and Cauffman 2021). These behavioral and cognitive changes happen while adolescents explore their environment and learn from myriad new life experiences as they increase in age, and this learning component plays an important role in cognitive development. This effect of life experience strengthens the association between age and behavioral/cognitive maturation during adolescence—an association that runs deep as several changes in brain structure and function also show age‐related patterns of maturation during this period (Galván 2021). However, this chronological improvement and its underlying learning processes are not all there is to this story. Adolescence is also the period of significant physiological maturation, most notably the pubertal transition (Lee and Styne 2013; Abreu and Kaiser 2016; Witchel and Topaloglu 2019), whose associated rising hormone levels can have potentially significant impact on brain and behavioral development.

In fact, some developmental changes in the brain seem to occur at least partially independently of chronological age. These changes have been associated with the time course of pubertal development, and include alterations in the patterns of brain activity and functional connectivity of several brain regions (Foulkes and Blakemore 2018; Goddings et al. 2019; Baum et al. 2020), dramatic synaptic plasticity processes leading to a net loss of synapses in early adolescence (synaptic pruning), and a progressive increase in myelination thereafter until early adulthood, which is particularly protracted in the prefrontal cortex (Huttenlocher and Debholkar 1997; Petanjek et al. 2011; Whitaker et al. 2016; Faria et al. 2021). All these changes vary from region to region in the central nervous system and are associated with the development of different cognitive abilities (Petanjek et al. 2011; Fuhrmann et al. 2015; Whitaker et al. 2016; Foulkes and Blakemore 2018; Baum et al. 2020; Faria et al. 2021; Galvan 2021).

The situation described supports the possibility that cognitive development during adolescence is affected by both age‐related effects of learning from experience, and puberty‐related effects on brain development. It is unclear, however, to what extent the level of cognitive maturation is determined by each of these effects. This can be difficult to ascertain because puberty‐related and age‐related effects are strongly correlated—after all, older adolescents tend to be more pubertally mature. Given this challenge, the goal of the present review is to assess the available evidence for the effects of pubertal status—that is, the stage of puberty an individual is in—*independent* of age‐related effects on a set of higher‐order cognitive capacities, namely, those that fall under the rubric of behavioral self‐regulation (SR).

### 1.1 The Maturation of Self‐Regulation and Executive Functions During Adolescence

Among the many cognitive changes occurring during adolescence, a particularly important set of alterations are those affecting the capacity to self‐regulate behavior. By SR we mean the abilities that enable flexible, adaptive, and goal‐directed control of behavior (Carver and Scheier 2001; Hofmann et al. 2012; Amaya 2020). This includes all capacities necessary to keep goals in mind and to implement, execute, and monitor behavioral strategies to achieve these goals (Carver and Scheier 2001; Hofmann et al. 2012; Amaya 2020). Of note, SR encompasses, but is broader than, self‐control. While the two terms are sometimes used interchangeably (e.g., Heatherton and Wagner 2011), self‐control is a more restricted construct and refers to the top‐down components of SR that resolve motivational conflicts and help override unwanted impulses and urges (Carver and Scheier 2001; Hofmann et al. 2012; Nigg 2017; Amaya 2020). As can be seen from its definition above, SR also encompasses the flexible and goal‐directed use of capacities traditionally placed under the rubric of executive functions (EFs).

While the precise definition of EFs is the subject of much debate, most definitions refer to capacities involved in flexibly and adaptively selecting, planning, executing, and monitoring goal‐directed behaviors (Baggeta and Alexander 2016; Garcia‐Barrera 2019; Bunge 2024). A good general definition is provided by Jurado and Rosselli (2007):

“[Executive functions] allow us to shift our mind set quickly and adapt to diverse situations while at the same time inhibiting inappropriate behaviors. They enable us to create a plan, initiate its execution, and persevere on the task at hand until its completion. Executive functions mediate the ability to organize our thoughts in a goal‐directed way.”

Most modern definitions of EF emphasize both its unity and diversity, with a variable set of functions being placed under the umbrella of EF, like specific tools in a general‐purpose toolbox (Baggeta and Alexander 2016; Garcia‐Barrera 2019; Bunge 2024). There are several taxonomies for the different functions that compose EF, but we will focus on a particularly useful distinction based on the behavioral context served by different functions. Specifically, EFs are important both to situations that do and that do not involve emotional or social stimuli/contexts, and the abilities important for these different contexts are commonly referred to as hot and cool EFs, respectively (Zelazo and Müller 2002; Hofmann et al. 2012; Baggeta and Alexander 2016; Nigg 2017). Cool EFs include abilities like controlled attention, inhibition of automatic responses, updating, shifting, planning, among others, all of which not involving socioemotional content or contexts (Friedman and Miyake 2017). Hot EFs, on the other hand, include abilities such as emotional regulation and the control of impulses under arousing and/or social conditions (Royall et al. 2002; Zelazo and Müller 2002; Hofmann et al. 2012; Baggeta and Alexander 2016; Nigg 2017; von Bastian et al. 2020).

It is important to note the significant overlap between definitions of SR and EF as presented in the previous paragraphs. In fact, we could say, based on our working definitions, that SR happens through EF, creating a functional identity between them. For that reason, we will mostly refer to these concepts together (i.e., “SR/EF”) for the remainder of this paper, only distinguishing between them when discussing cognitive tests or questionnaires traditionally associated with one or the other term—but always keeping in mind that these constructs are deeply intertwined.

Particularly important to our focus on adolescent cognitive development is the fact that successful SR/EF involves a number of brain regions acting in coordination. However, some of these regions mature earlier, while others show a protracted developmental course, with a lot of nonlinear trajectories (Ernst et al. 2006; Heatherton and Wagner 2011; Ernst 2014; Casey 2015; Casey and Caudle 2013; Shulman et al. 2016; Gracia‐Trabuenca et al. 2021; Icenogle and Cauffman 2021). Therefore, the precise developmental course of SR/EF development may vary depending on the maturation of the brain systems that subserve the distinct abilities included in this broad construct.

There are different theoretical perspectives that attempt to explain the maturation of SR/EF during adolescence, often focused on specific aspects of adolescent cognitive development, such as social behavior (Nelson et al. 2016) and decision‐making under risk and uncertainty (Defoe et al. 2015; Romer et al. 2017). But there is one particularly prominent set of models that attempt to explain broader aspects of adolescent SR/EF maturation, one that is generally referred to as imbalance models, which include dual systems and the triadic models (for reviews, see Ernst et al. 2006; Ernst 2014; Casey 2015; Shulman et al. 2016). Of these, the dual‐system models are the most widely considered by the literature.

Dual‐system models vary in some respects but generally propose that, on the one hand, from childhood to adulthood, cool SR/EF show an overall linear improvement (Yurgelun‐Todd 2007; Steinberg et al. 2008; Crone 2009; Crone and Steinbeis 2017; Steinberg et al. 2018; Gabriel et al. 2021; Icenogle et al. 2019; Icenogle and Cauffman 2021), or an initial improvement with a midadolescence plateau (Luna and Wright 2016; Constantinidis and Luna 2019; Icenogle and Cauffman 2021), which accompanies the maturation of frontal brain regions.

On the other hand, these models suggest a nonlinear developmental trajectory for the socioemotional brain systems including the ventral striatum and adjacent structures (Ernst 2014; Shulman et al. 2016), which are associated with approach/reward behaviors and are thus important for hot SR/EF. The developmental trajectory of these regions is believed to follow an inverted‐U‐shaped curve, with their activity peaking at some point in midadolescence and then falling as they come under stronger control from prefrontal regions, creating a transitory period in which individuals can find it harder to self‐regulate socioemotional and reward‐seeking behaviors (Heatherton and Wagner 2011; Shulman et al. 2016; Icenogle and Cauffman 2021). Of note, other nonlinear trajectories have also been proposed (Casey 2015, 2015; Casey and Caude 2013), but the proposed transitory hot SR/EF issues remain. In all cases, the idea is that the frontal systems are transiently incapable of reigning in the affective systems, causing adolescents to have difficulties in restraining impulses and regulating emotion under arousing, motivational, and/or social situations, predisposing them to reckless behavior (Steinberg 2008; Heatherton and Wagner 2011; Smith et al. 2013; Luna et al. 2015; Shulman et al. 2016; Steinberg et al. 2018; Icenogle and Cauffman 2021).

Importantly, the view of adolescent risk‐taking as reflecting SR/EF failures in adolescence is criticized by other models, which propose more nuanced mechanisms and/or alternative interpretations of these behaviors (for reviews, see Nelson et al. 2016, 2014; Defoe et al. 2015; Romer et al. 2017; França and Pompeia 2024). A particularly important point of contention lies in the relationship between SR/EF and risky behavior, which can be complex and is not always clearly captured by the instruments used to measure risk‐taking. For example, questionnaires used to inquire about real‐world risk‐taking behaviors often focus on the occurrence and frequency of risky behaviors without directly assessing whether those behaviors were indeed products of failures in SR/EF (see Box 1 for further discussion of this issue and its implications for this review). Despite these conceptual difficulties, the dual‐systems perspective on the development of SR/EF and related capacities during adolescence remains widely accepted (Shulman et al. 2016; Icenogle and Cauffman 2021).

**Box 1: Risk‐taking, sensation seeking, and their relation to self‐regulation/executive function**

A notable absence in this review is that of studies employing many types of measures usually regarded as assessing risk‐taking, sensation seeking, and related constructs such as reward sensitivity. While these constructs are usually associated with self‐regulation/executive function (SR/EF)—and, more specifically, to self‐control (e.g., de Ridder et al. 2011; Robson et al. 2020)—the constructs themselves and the instruments used to measure them do not necessarily assess the capacities for SR/EF or self‐control as defined in our study.

Let us start with risk‐raking questionnaires. Many (though not all) behaviors commonly considered as risk‐taking, such as alcohol and drug use, and sexual activity, should not be automatically regarded as failures of SR/EF or self‐control. Adolescents may deliberately choose to engage in these activities and even go to great lengths to do so. Thus, the goals of the adolescents themselves in engaging in a behavior should be taken into account, not just its potential outcome (Do et al. 2020; França and Pompeia 2024). Furthermore, to consider a behavior as a failure of SR/EF and a “risk‐taking” behavior implies that the individual fully appreciates the risks involved (Jessor 2018), which may not be the case because adolescents can lack knowledge and experience to do so. Also, it cannot be disregarded that at least part of the variability in “risky” behaviors reflect differences in risk‐exposure—that is, in the opportunity to take such risks—not in risk‐taking (Defoe et al. 2015, 2019), as the former tends to increase as adolescents grow older because of progressive reduction of parental supervision.

According to Romer et al. (2017), decision‐making differs depending on risk probabilities. There are many different types of risk‐taking, only some of which are maladaptive and may peak during adolescence. This phase of life is marked by behaviors associated with sensation seeking and impulsive action that are specifically motivated to allow the exploration of the environment. But these risky behaviors are only more prevalent in adolescents than in adults and children under conditions in which risk outcomes are uncertain or ambiguous; when the risks are unambiguous, that is, when the outcomes can be clearly calculated (e.g., both involve sure wins of different magnitudes), adolescent behavior is midway between that of children and adults, indicating that the SR/EF abilities involved in this type of decision‐making develop linearly without a peak in risk‐taking during adolescence (Romer et al. 2017). Some individuals, however, do seem to be insensitive to risk. They present high levels of acting without thinking or difficulties with self‐regulatory impulse control that precedes adolescence, increases in adolescents, and remains elevated in adulthood (Romer et al. 2017). Therefore, risk‐taking/impulsive action in adolescence has many possible interpretations that essentially depend on the type of choice the adolescent must make. Determining failures of SR/EF in conditions involving risks and benefits should ideally be done considering whether choices involve actual risks, the uncertainty around the probabilities involved, and the ability of adolescents of appreciating the risks. Furthermore, questionnaires that address *self‐rated* SR/EF failures in ecologically valid circumstances (i.e., in real life) are particularly relevant for determining how pubertal status affects behavior during adolescents’ daily lives, as these could be constructed in such as way allow distinguishing behaviors that were indeed caused by failures of SR/EF (França and Pompeia 2024).

In light of these considerations, we adhered to the following rationale in this review: In tasks involving risk‐taking, if the results are to be used as an index of SR/EF, the task must have a strategic component that requires the capacity to choose the best course of action—and the performance in the task must assess whether or not the test‐taker succeed in doing so. To illustrate this point, consider the Balloon Analogue Risk‐Taking task (BART; Lejuez et al. 2007), and its younger cousin, the Balloon Risk‐Avoidance Task (BRAT; Crowley et al. 2021). Both these tasks can involve strategic decision‐making under uncertainty, where participants gain points by progressively inflating or deflating virtual balloons through button presses, but lose all points if the balloon burst, which occurs at unexpected levels of balloon total size. The ideal performance metric must reflect a balance between inflating the balloon as much as possible (in the BART), or deflating it as little as possible (in the BRAT), while avoiding explosions, so that “risk‐takers” or “impulsive” individuals should end up with less points than people who are more cautious/less impulsive. However, many studies with adolescents (e.g., Collado et al. 2014; Loman et al. 2014; Crowley et al. 2021) do not take into account the number of balloons that burst or the participants’ final earnings. Instead, they use scores like the total number of balloon pumps independently of whether that balloon burst, which does not reflect how participants fare when trying to balance earning more points with the possibility of bursting the balloons. Consequently, these performance variables do not index SR/EF or related capacities.

As for sensation seeking and reward sensitivity, instruments that assess these constructs often evaluate something that fits more into the category of personality traits or “tastes and preferences” than in the category of “SR/EF abilities.” Examples include questions such as “I would like to learn to fly an airplane,” from the widely used Zuckerman Sensation Seeking Scale‐V (Zuckerman et al. 1978), or the item “I like new and exciting experiences, even if I have to break the rules,” from the Brief Sensation Seeking Scale–4 (Stephenson et al. 2003). We and others (e.g., Romer et al. 2017) consider that people who actively search for sensations and rewards, and who may employ their cognitive capacities toward the attainment of these goals, should not be regarded as having SR/EF difficulties. For these reasons, studies examining the effects of pubertal development on scores of tasks/questionnaires with the characteristics above were not included in this review.

Regardless of the extent to which SR/EF failures are to blame for real‐world risk‐taking behaviors, SR/EF are clearly necessary for reaching analytic, rational, nonemotional goals (cool SR/EF), and are positively associated with important real‐life outcomes, like educational attainment, professional success, and wealth (e.g., Moffitt et al. 2011; Eisenberg et al. 2019; Benjamin et al. 2020; Robson et al. 2020). Furthermore, at least some SR/EF difficulties that involve socioemotional stimuli and/or contexts (i.e., hot SR/EF) have also been found to be negatively associated with mental and physical health throughout life, such as self‐reported poor emotional control, and impulsivity (e.g., Moffitt et al. 2011; Eisenberg et al. 2019; Benjamin et al. 2020). Therefore, there is great interest in understanding how both cool and hot SR/EF mature during adolescence, a period that may provide an ideal window for intervention, thanks to the heightened plasticity of the brain in this phase of life (Dorn et al. 2019).

### 1.2 Puberty and Cognitive Development During Adolescence

The development of SR/EF has been most commonly studied in relation to the individuals’ age or related variables, such as school grade. The age variable is commonly used to represent general maturation and, in the context of cognitive development, it is also viewed as a proxy for the accumulation of life and academic experiences. Thus, age works as an aggregating variable in developmental research, loosely capturing the results of a wide range of developmental processes that take place as individuals grow older. However, the age variable does not allow us to disentangle the effects of these different developmental processes, and some of them may be more important than others (McLean and Riggs 2022). A particularly relevant process taking place during adolescence—and one that may have important effects on the development of SR/EF—is puberty.

Puberty is a developmental process involving a cascade of neuroendocrine events that lead to the attainment of reproductive capacity (Lee and Styne 2013; Abreu and Kaiser 2016; Witchel and Topaloglu 2019). More specifically, puberty is characterized mainly by the effects of increased production of androgens by the zona reticularis of the adrenal glands, and a surge of gonadal hormones due to the activation of the hypothalamic–pituitary–gonadal axis. These adrenal and gonadal hormones then go on to induce, in concert with other hormones, a series of morphological and physiological changes, including growth in height, maturation of the gonads, and the development of secondary sexual characteristics (DiVall and Radovick 2009; Lewis and Lee 2009; Wood et al. 2019).

Importantly, pubertal processes also influence structural and functional brain changes implicated in SR/EF (Casey 2013; Casey and Caudle 2013; Peper and Dahl 2013; Juraska and Willing 2017; Foulkes and Blakemore 2018; Goddings et al. 2019; Gracia‐Tabuenca et al. 2021). Animal studies even raise possible mechanisms behind the relationship between puberty and brain maturation, such as through the effects of estrogen on the inhibitory/excitatory balance in frontal brain regions (Piekarski et al. 2017), or through hormonal effects on the production of new brain cells during adolescence (Ahmed et al. 2008). However, in humans, many questions remain as to the precise contribution of the pubertal transition to changes in different brain regions during adolescence (Goddings et al. 2019), and their relation with behavioral and cognitive changes (Ernst et al. 2006; Yurgelun‐Todd 2007; Crone 2009; Pfeifer and Allen 2012; Smith et al. 2013; Ernst 2014; Casey 2015; Murty et al. 2016).

Despite these open questions, the available evidence suggests that, at any given moment during the life of an adolescent, his or her SR/EF abilities may reflect not only their age, but also the stage of puberty they are in (i.e., their pubertal status). Crucially, pubertal status can vary significantly between individuals as there is great variability in the age at which puberty starts (i.e., the absolute pubertal timing^1^), and in its developmental course (i.e., the pubertal tempo; Dorn et al. 2006; Dorn 2006, 2015; Huang et al. 2009; Dorn and Biro 2011; Mendle et al. 2019). For example, it is considered normal for girls to show the first physical signs of puberty (i.e., to “enter puberty”) between 8 and 13 years of age, while boys do so around the ages of 9–14 years; the total time taken to reach the end of puberty in both sexes also varies widely (Dorn et al. 2006; Dorn and Biro 2011; Joos et al. 2018; Mendle et al. 2019), spanning from around 2 to 6 years (Joos et al. 2018), although most adolescents are regarded as pubertally mature at around the age 16 years (see Marceau et al. 2011).

The recognition of these interindividual changes in terms of age of onset and end of puberty, as well as its nonlinear trajectory, has contributed to the growing interest in exploring pubertal effects on cognition and behavior in the last few decades (Marceau et al. 2019). During this period, many recommendations have been proposed regarding ways to incorporate pubertal measures in developmental studies (Dorn et al. 2006; Huang et al. 2009; Dorn and Biro 2011; Dorn 2015; Byrne et al. 2019; Mendle et al. 2019; Cheng et al. 2020). However, conceptual difficulties remain. There are significant variations in the synchronicity of different puberty‐induced changes, with only small‐to‐moderate correlations between different physical developmental makers such as pubic hair growth and breast/genital development, and lack of a clear match between physical changes and sex hormone concentrations (Dorn and Biro 2011; Mendle 2014; Mendle et al. 2019; França et al. 2022). Therefore, the choice of variables that can best represent each individual's pubertal status can be a difficult one.

**FIGURE 1:**
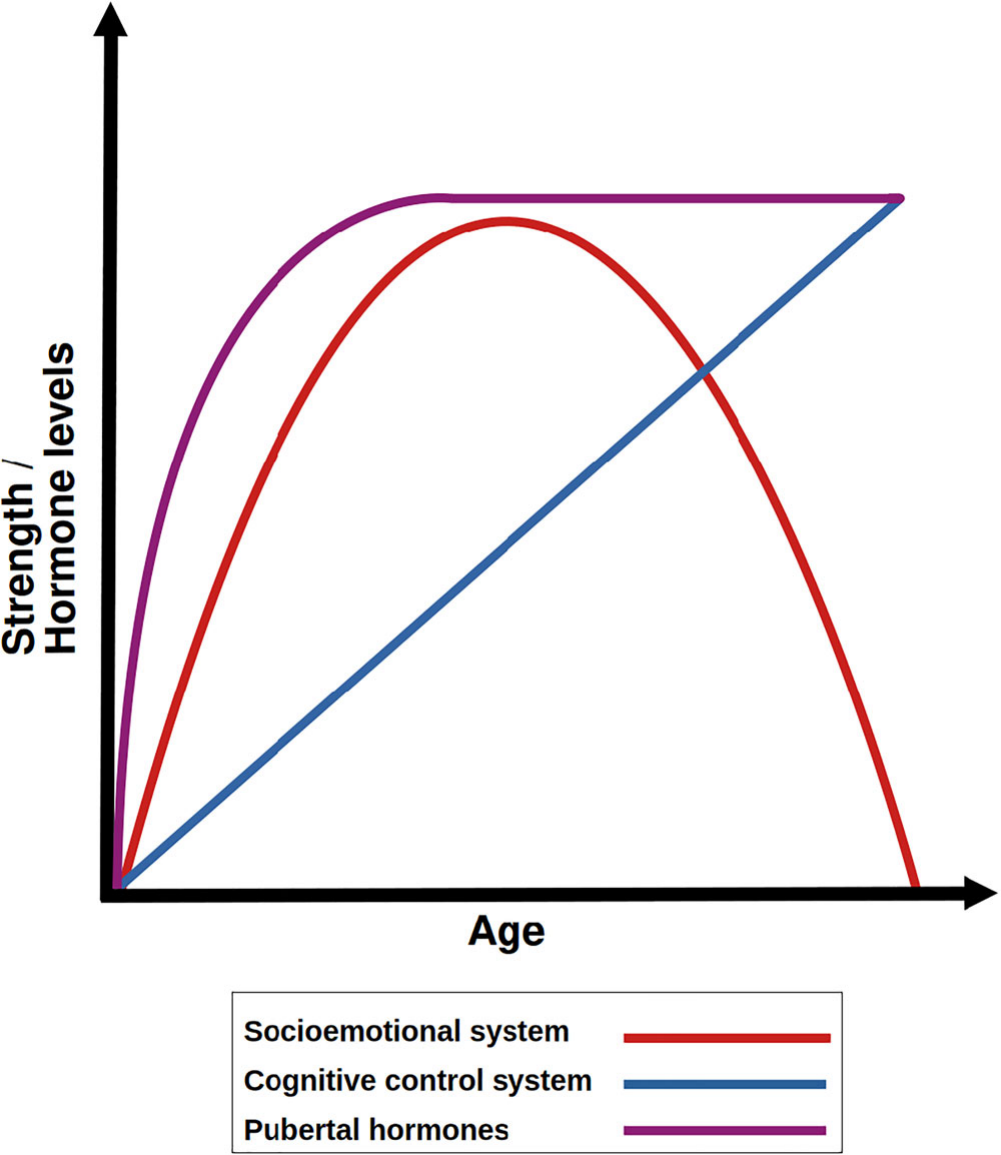
A simplified illustration of one of the dual‐system models, based on the model endorsed by Shulman et al. (2016). The graph shows the general shape of the hypothesized trajectories of development of the two systems during adolescence, from roughly around age 10 to age 25. This includes a linear trajectory for cognitive control system (based on the development of frontal regions, independent of pubertal development), and a nonlinear, inverted‐U‐shaped trajectory for the socioemotional system (based on the development of subcortical dopamine‐rich regions), which is initially sensitive to the increase in gonadal hormones during puberty, but gradually desensitizes. Of note, variations of the dual systems sometimes propose different trajectories for either the cognitive control system or the socioemotional control system, but they are consistent in their claim of some sort of transient imbalance between the two systems, with pubertal hormones affecting mainly the latter and not the former.

Moreover, although the above‐mentioned models of adolescent cognitive development consider that part of the changes in SR/EF are associated with puberty (e.g., Ernst et al. 2006; Ernst 2014; Shulman et al. 2016), they often do not explore this issue in depth, particularly the impact of variability in pubertal onset and progression in creating variability in SR/EF performance within same‐aged adolescents. Nonetheless, proponents of these models suggest that the prefrontal changes that support cool SR/EF develop independently of pubertal status, with the most cited models positing a linear pattern of improvement throughout adolescence (although this hypothesis is challenged by evidence from animal studies [Piekarski et al. 2017] showing effects of sex hormones on frontal brain regions during puberty). On the other hand, these models posit that cognitive processes that involve hot, socioemotional networks are dependent on puberty, peaking in midadolescence due to increasing levels of gonadal hormones. The idea is that the rise in gonadal hormones affects subcortical regions involved in reward‐processing, particularly those related to the mesolimbic dopaminergic pathway, leading to a corresponding peak in impulsive reward‐seeking, risk‐taking behaviors, that eventually subsides as the sensitivity of those regions to gonadal hormones gradually reduces (Figure 1; Ernst et al. 2006; Ernst 2014; Shulman et al. 2016; but see França and Pompeia (2024) for a critical review of the role of dopamine in adolescent risky behaviors). Of note, the existence of this proposed midadolescence peak in risky behaviors has been questioned (see Defoe et al. 2015), but the general idea of differential pubertal effects on hot and cool SR/EF has remained (Shulman et al. 2016).

In short, imbalance models pose no pubertal effects on cool SR/EF, along with nonlinear effects of pubertal status on hot SR/EF, with pubertal development initially leading to worse hot SR/EF performance, which then reverses as development progresses. The precise trajectories of the development of the cognitive control and socioemotional systems vary depending on the particular version of the dual‐systems model (Casey 2013; 2015; Casey and Caudle 2013; Shulman et al. 2016; Icenogle and Cauffman 2021), but their behavioral predictors and the proposed effect of pubertal development are very similar between them. Of note, based on these models, we could also expect differential effects of earlier or faster pubertal maturation (i.e., earlier timing and faster tempo) on hot SR/EF, given that the proposed trajectory of cognitive effects is mediated by the rise of hormones and subsequent changes in the brain's sensitivity to them. While these variations may or may not be interpretable in terms of variations in pubertal status, they are not explored in detail in discussions of imbalance models and will not be the focus of this review.

Determining the relationship of puberty with SR/EF is a particularly thorny issue because of the difficulty in disentangling the effects of pubertal status from the effects of age, as both are correlated, despite the large interindividual differences in terms of the pubertal trajectory. Stated differently, older individuals tend to be, on average, more advanced in their pubertal development (Cheng et al. 2020), so the relation between pubertal status at the time SR/EF outcomes are assessed might merely index age effects (like, e.g., improvements in SR/EF due to gains in life experience). It is therefore paramount to understand whether, and to what extent, pubertal status affects adolescents’ ability to self‐regulate behavior beyond the effects of their age, regarding both hot and cool skills.

These issues have implications for academic contexts and for policy‐making. For example, the school system is based on the understanding that adolescents who are the same age should display comparable cognitive abilities, despite the fact that adolescents in the same grade can be at very different stages of puberty and thus differ in terms of their brain and cognitive maturation. There is evidence that higher pubertal maturity is positively related with academic success (Torvik et al. 2021), although findings from studies investigating such association are not always clear‐cut (Laube and Fuhrmann 2020; Suutela et al. 2022), and there seem to be important sex differences, with earlier maturation in girls being associated with poorer academic performance (Cavanagh et al. 2007; see also results for age at menarche in Torvik et al. 2021). Such associations are further complicated by social factors, such as differences in teachers’ expectations being influenced by both relative pubertal timing and ethnicity (Carter et al. 2018). Despite current gaps in our knowledge, it is clear that pubertal development cannot be ignored when discussing academic performance. Similarly, policy‐making regarding adolescents could also profit from considering pubertal status beyond age in terms of maximizing preventive socioeducational measures to decrease adolescents’ vulnerability, impulsive actions and risk‐taking, and improve their health and well‐being, which involve improving SR/EF development, as well as building a fairer legal system regarding adolescents’ rights, duties, and accountability (Galván 2014; Dorn et al. 2019).

### 1.3 The Present Study

In this scenario, our primary goal was to systematically assess the evidence regarding the association of pubertal *status* with SR/EF, independent of age effects, in typically developing adolescents (i.e., nonclinical samples). By pubertal status, we mean the stage of sexual development an individual was in *at the time* his or her SR/EF abilities were assessed, independent of their age, the age when they entered puberty, and/or their rate of pubertal progression. Therefore, we reviewed studies that concurrently assessed SR/EF and any indicator of pubertal status (be it physical characteristics or concentrations of substances like sex hormones) and that reported their association.

Here, we must raise three important points. First, because we were interested in the effects of pubertal status—the stage of development the individual is in when behavior was assessed—, we did not review studies reporting effects of absolute pubertal timing (the age at which pubertal changes first became apparent), relative pubertal timing (whether pubertal development was earlier or later compared to adolescents’ same age/sex peers, regardless of their actual pubertal status) or tempo (rate of the pubertal trajectory), as discussed in more detail in the Methods section.

Second, we intended to describe the effects of pubertal status on SR/EF that cannot be attributed to differences in age, so we focused on studies that took into consideration the effects of age as a continuous variable (age in months or noninteger years) in the statistical analyses (see Cheng et al. 2020). This was done because there is extensive evidence that pubertal status can change within a period of one full year (for a clear illustration, see data in Gracia‐Trabuenca et al. 2021) and that there are cognitive differences between older and younger individuals when age is counted in completed years, even within the same school grade (e.g., Peña 2020).

Third, while we have a specific definition for SR/EF, there are many different (and sometimes conflicting) definitions in the literature. As a result, there are several available tasks and scales that measure SR/EF‐related skills (Zelazo and Müller 2002; Chan et al. 2008; Baggeta and Alexander 2016; Nigg 2017), with a many‐to‐many mapping between definitions and instruments, and frequent jingle–jangle fallacy issues (e.g., Packwood et al. 2011). We only review studies whose measures fit the definition of SR/EF used in the present review (as discussed earlier, with more details on this issue provided in the Methods section).

In what follows, we present our approach to reviewing the literature and provide a critical account of our findings. As described in detail below, we found that most available studies reporting results on the relationship between measures of pubertal status and SR/EF did not adjust for age effects, or did so in ways that do not enable an adequate interpretation of the results in terms of pubertal status effects. We also show that available studies on the effects of pubertal status on SR/EF used a variety of different (and often difficult to compare) measures of pubertal status and SR/EF. These studies show inconsistent results, with no clear patterns, and their reliability is limited by a set of common methodological pitfalls ubiquitous in this and related fields. We conclude by providing suggestions for addressing these issues and improving the level of information in future studies.

## 2 Methods

For designing the review, we took guidance from Cooper (1982), Jackson (1980), Higgins and Green (2008), Gough et al. (2012), Leenaars et al. (2012), Bramer et al. (2017), and Siddaway et al. (2019). For reporting the review results, we followed the PRISMA 2020 guidelines whenever applicable (Page et al. 2021). Next, we will briefly describe the review process, highlighting changes to our original, preregistered protocol, which is available online (http://doi.org/10.17605/OSF.IO/URMBX). Additional information can be found on the project's page at the Open Science Framework (OSF; https://doi.org/10.17605/OSF.IO/XFNUZ).

### 2.1 Eligibility Criteria

Our research question was: “What is the effect of pubertal status on SR/EF in healthy, typically developing adolescents?”. To be relevant to our research question, studies included in this review needed to have certain characteristics in terms of their design, the population under study, and the measures employed. Specifically, we looked for studies, in any language, with the following characteristics:

- **Study design**: We included studies reporting original empirical results. They could be either longitudinal or cross‐sectional studies evaluating SR/EF during the pubertal transition.
- **Participants**: Studies had to involve human participants at any age between 10 and 19 years, following the definition of adolescence by the World Health Organization (“Health for the world's adolescence” report: Dick and Fergusson, 2015). Importantly, this age range was not implemented as a rigid age limit for excluding studies—studies that also included participants younger or older than our specified age range could be included, as long as the study dealt with our review question. Additionally, studies should focus on nonclinical samples to exclude the interference of clinical conditions that can influence SR/EF and/or pubertal development. Hence, we did not include studies on clinical populations, such as with developmental disorders (e.g., attention deficit hyperactivity disorder, learning disabilities, and autism spectrum disorder), mental health problems, addiction, eating disorders, and pathological pubertal development (e.g., central or peripheral precocious or delayed puberty), except if they used nonclinical control groups for which results fitting the criteria of this review were reported.
- **Measurements of pubertal status**: We only included in the review studies that employed at least one method to evaluate pubertal status, such as Tanner Stages, the Pubertal Development Scale (PDS), hormonal assays (see Dorn et al. 2006; Dorn and Biro 2011; Mendle et al. 2019), or any other measure based on physical or physiological markers of puberty.
- **Measurements of SR/EF**: Studies should also report at least one suitable measure of SR/EF. Eligible measures of SR/EF could be either laboratory tasks or questionnaires/scales. These measures should assess outcomes directly related to SR/EF abilities—that is, with abilities required for flexible, adaptive, and goal‐directed control of behavior. This criterion was relatively straightforward for tasks, and most of the tasks traditionally associated with behavioral SR and EF were considered. We excluded measures that predominantly involve so‐called crystallized intelligence (e.g., receptive vocabulary and general knowledge), because they assess cognitive abilities such as language and semantic memory, which are highly dependent on parental schooling (e.g., Cheadle 2009) and academic experience, and not *primarily* related with SR/EF as defined here. However, measures of fluid intelligence (e.g., block design and various forms of matrices tests) were included, as they are regarded as highly associated with cool SR/EF (Royall et al. 2002; Zelazo and Müller 2002; Chan et al. 2008). Applying this criterion to questionnaires was more difficult, because many questionnaires that are traditionally associated with SR/EF do not fit our definition of this construct. For example, scales that investigate problem/risk behaviors, reward sensitivity, and sensation seeking do not usually differentiate behaviors that were carried out due to SR/EF failures from those involving deliberate choices made by adolescents (see França and Pompeia 2024 for a detailed discussion of this issue). This is the case of many scales that regard drinking alcoholic beverages, or enjoying radical sports in adolescence as SR/EF failures, without considering that individuals often can (and do) purposefully/deliberately choose to engage in such behaviors, in which case the behavior should not be considered a SR/EF failure (we provide further discussion about this issue in Box 1). Based on this criterion, most included questionnaires were based on self‐reported items of hot or cool SR/EF failures or difficulties.
- **Analyses of the relationship between pubertal status and relevant outcomes**: For inclusion in the review, studies also had to report results on the statistical relationship between the measure of pubertal status and the measure of SR/EF obtained in close proximity in time (no more than a few days apart). This criterion is important because we found many studies employing these measures but that did not report results on their relationship, as well as a few studies that measured puberty and SR/EF with several months or even a year of difference.
- **Controlling/adjusting for age effects**: Finally, studies had to control or adjust for the effects of age in such a way that results could be interpreted as reflecting the association (or lack of) of pubertal status and SR/EF that could not be explained by age‐related SR/EF improvement. Age should be reported in years with at least one decimal point or in months because, as mentioned in the Introduction section, both pubertal status and cognitive abilities advance within a 1‐year period during this phase of life.

### 2.2 Information Sources and Search Strategy

To find relevant studies, we searched PubMed, Web of Science (Core Collection), Scopus, and PsycINFO (via APA). The search strings used in these databases were built around the two main concepts in our research question, namely, “puberty” and “constructs related to SR/EF.” Building a sensitive search string for the concept of SR/EF was challenging, and to find relevant terms we consulted literature reviews (e.g., Zelazo and Müller 2002; Chan et al. 2008; Baggeta and Alexander 2016; Nigg 2017), the thesaurus of PubMed and PsycINFO (APA), and had the input of field experts (authors Sabine Pompeia and Mônica C. Miranda). After several rounds of testing and evaluating search results for relevance, we ended up with the following list of terms:

- Concept #1 (puberty): Puberty; Pubertal.
- Concept #2 (SR/EF of behavior and executive functions): “SR/EF”; “self‐control”; “behavio* control”; “behavio* regulation”; “regulation of behavio*”; “control of behavio*”; “executive function*”; “central executive”; “executive control”; “executive network”; “inhibitory control”; cogniti*; “working memory”; intelligence; attention*; “decision‐making”; “decision‐making”; “academic achievement”; “academic success”; “emotion* regulation”; “emotion* control”; “regulation of emotion”; “control of emotion”; “theory of mind”; “facial recognition”; “facial expression*”; reward; “sensation seeking”; “risk taking”; “risk‐taking”; impulsiv*; “impulse control”; “novelty seeking”; “novelty‐seeking”; “delayed discounting”; “temporal discounting.”

As already noted in the Introduction section, there are conflicting definitions of SR/EF in the literature and the relationship between measures and concepts can be complex. For this reason, irrespective of the terminology used by the authors of the reviewed papers to describe which cognitive/behavioral ability was being tested, we opted to work with a broad range of measures that are usually associated with SR/EF (e.g., controlled attention, inhibition, self‐control, impulsiveness). Hence, the decision on whether or not to include the study in this review was carried out case‐by‐case during the evaluation of full texts based on the description of the SR/EF tasks or questionnaires. We return to this issue in more detail when describing the criteria used to select these measures.

The search terms were used to build search strings according to the syntax of each search engine. The final search strings used for each engine are presented in Table 1. The searches were originally conducted in February 2020, and were last updated in February 21st, 2024. To look for gray literature, we contacted the corresponding authors of included studies via email (when emails were available in the respective papers) asking for any additional studies they may have conducted on the topic, published or not.^2^ We also refrained from using filters for document types on the search engines to include any theses, dissertations, or congress/symposium‐related publications indexed in the databases searched.

**TABLE 1:** Search strings used for each search engine.

Search engine	Search string
**PubMed**	((“Puberty”[Mesh] OR “Puberty”[tiab] OR “Pubertal”[tiab]) AND (“Executive Function”[Mesh] OR “Self‐Control”[Mesh] OR “Emotional Regulation”[Mesh] OR “Problem Solving”[Mesh] OR “Attention”[Mesh] OR “Inhibition, Psychological”[Mesh] OR “Intelligence”[Mesh] OR “Cognition”[Mesh] OR “Decision Making”[Mesh] OR “Theory of Mind”[Mesh] OR “Impulsive Behavior”[Mesh] OR “Emotional Intelligence”[Mesh] OR “Risk‐Taking”[Mesh] OR “Facial Expression”[Mesh] OR “Facial Recognition”[Mesh] OR “Delay Discounting”[Mesh] OR “Reward”[Mesh] OR “Academic Success”[Mesh] OR “Self‐regulation”[tiab] OR “self‐control”[tiab] OR “behavioral control”[tiab] OR “behavioural control”[tiab] OR “behavioural regulation”[tiab] OR “behavioral regulation”[tiab] OR “regulation of behavio*”[tiab] OR “control of behavio*”[tiab] OR “executive function*”[tiab] OR “central executive”[tiab] OR “executive control”[tiab] OR “executive network”[tiab] OR “inhibitory control”[tiab] OR “cogniti*”[tiab] OR “Working memory”[tiab] OR “Intelligence”[tiab] OR “attention*”[tiab] OR “decision making”[tiab] OR “decision‐making”[tiab] OR “academic achievement”[tiab] OR “academic success”[tiab] OR “emotion regulation”[tiab] OR “emotional regulation”[tiab] OR “emotion control”[tiab] OR “emotional control”[tiab] OR “regulation of emotion”[tiab] OR “control of emotion” OR “theory of mind”[tiab] OR “facial recognition”[tiab] OR “facial expression*”[tiab] OR “reward”[tiab] OR “sensation seeking”[tiab] OR “Risk taking”[tiab] OR “risk‐taking”[tiab] OR “impulsiv*”[tiab] OR “impulse control”[tiab] OR “novelty seeking”[tiab] OR “novelty‐seeking”[tiab] OR “delayed discounting”[tiab] OR “temporal discounting”[tiab]))
**Web of Science**	TS = ((puberty OR pubertal) AND (“self‐regulation” OR “self‐control” OR “behavio* control” OR “behavio* regulation” OR “regulation of behavio*” OR “control of behavio*” OR “executive function*” OR “central executive” OR “executive control” OR “executive network” OR “inhibitory control” OR cogniti* OR “working memory” OR intelligence OR attention* OR “decision‐making” OR “decision‐making” OR “academic achievement” OR “academic success” OR “emotion* regulation” OR “emotion* control” OR “regulation of emotion” OR “control of emotion” OR “theory of mind” OR “facial recognition” OR “facial expression*” OR reward OR “sensation seeking” OR “risk taking” OR “risk‐taking” OR impulsiv* OR “impulse control” OR “novelty seeking” OR “novelty‐seeking” OR “delayed discounting” OR “temporal discounting”))
**Scopus**	TITLE‐ABS‐KEY((“puberty” OR “pubertal”) AND (“self‐regulation” OR “self‐control” OR “behavio* control” OR “behavio* regulation” OR “regulation of behavio*” OR “control of behavio*” OR “executive function*” OR “central executive” OR “executive control” OR “executive network” OR “inhibitory control” OR “cogniti*” OR “working memory” OR “Intelligence” OR “attention*” OR “decision‐making” OR “decision‐making” OR “academic achievement” OR “academic success” OR “emotion* regulation” OR “emotion* control” OR “regulation of emotion” OR “control of emotion” OR “theory of mind” OR “facial recognition” OR “facial expression*” OR “reward” OR “sensation seeking” OR “risk taking” OR “risk‐taking” OR “impulsiv*” OR “impulse control” OR “novelty seeking” OR “novelty‐seeking” OR “delayed discounting” OR “temporal discounting”))
**PsycINFO**	((puberty OR pubertal) AND (“self‐regulation” OR “self‐control” OR “behavio* control” OR “behavio* regulation” OR “regulation of behavio*” OR “control of behavio*” OR “executive function*” OR “central executive” OR “executive control” OR “executive network” OR “inhibitory control” OR cogniti* OR “working memory” OR intelligence OR attention* OR “decision‐making” OR “decision‐making” OR “academic achievement” OR “academic success” OR “emotion* regulation” OR “emotion* control” OR “regulation of emotion” OR “control of emotion” OR “theory of mind” OR “facial recognition” OR “facial expression*” OR reward OR “sensation seeking” OR “Risk taking” OR “risk‐taking” OR impulsiv* OR “impulse control” OR “novelty seeking” OR “novelty‐seeking” OR “delayed discounting” OR “temporal discounting”))

### 2.3 Study Selection

The study selection was conducted in two stages. First, titles and abstracts were screened. In stage 2 we evaluated the full texts of approved studies in stage 1 and studies that could not be eliminated with certainty based on consideration of the title and abstract. At both steps, every record/study was assessed by two investigators independently (Thiago F. A. França and either Sabine Pompeia, Mônica C. Miranda, Isis A. Segura, Natália M. Dias, or one of two trained research assistants). During study selection, we used the following inclusion criteria, assessed in this particular order:

1. Report original empirical results;
2. Report data for nonclinical populations assessed at any age between 10 and 19 years (although this age range was flexible, as discussed above);
3. Use of at least one measure of pubertal status as defined above;
4. Use of at least one cognitive/behavioral measure of SR/EF as conceptualized based on the literature described in the Introduction section;
5. Report results regarding the statistical relationship between pubertal measures and cognitive outcomes of interest;
6. Report results that are interpretable in terms of the direct association of pubertal status and SR/EF abilities. This implied that these measures had to be obtained in close proximity in time and had to be controlled/adjusted for age effects in a way that did not affect the interpretation of pubertal variables (i.e., not creating new variables with different meanings as happens in studies focusing relative pubertal timing; see the **Results** section for more details). Of note, this criterion was only evaluated at the second stage of study selection.

Criteria #5 and #6 on the list above were not originally made explicit in the preregistered protocol, but were added here because our primary objective of investigating the relationship between pubertal status and SR/EF could only be reached if these precepts were kept. Without these criteria we would have ended up with studies that measured pubertal status and SR/EF but did not report their association, and/or studies that measured them at different times, and/or converted pubertal status measures into measures of relative pubertal timing, which do not speak to their direct relationship. In fact, there was an unexpectedly large number of studies measuring pubertal status and SR but that did not report their relationship (see details in the Results section). Among these studies were those that measured pubertal status only to make sure that study groups did not differ significantly with regard to this variable (e.g., Jarcho et al. 2016; Bartholdy et al. 2019; Bernardes et al. 2020), to guarantee the inclusion of only pre‐, mid‐, or postpubertal participants in the study (e.g., Ludyga et al. 2019), or just to characterize the sample (e.g., Pilhatsch et al. 2014; Kessler et al. 2017; Vetter et al. 2013). Other studies used pubertal development measures to adjust the statistical analyses for variables that were not related to SR (e.g., Lauzon‐Guillain et al. 2006; Gaysina et al. 2013; Squeglia et al. 2014; Schmidt et al. 2015; Whalen et al. 2015; Adeli et al. 2020; Richmond‐Rakerd et al. 2020; Ruotsalainen et al. 2020; Adise et al. 2021), to assess whether pubertal status mediates/moderates effects that were not of interest to this review (e.g., Cabral et al. 2021; Fuhrmann et al. 2021), to assess the effects of both pubertal development and SR on some other variable of interest (Markey et al. 2003; Tarter et al. 2007; Kong et al. 2013; Kelly et al. 2016; Ozkan and Worrall 2017; Thompson et al. 2017; Ahmed et al. 2020; Porcelli et al. 2023), or included brain imaging findings while performing cognitive tasks without reporting/analyzing the actual performance on the task in relation to pubertal status (e.g., Gracia‐Tabuenca et al. 2021). There were also studies that built composite scores including SR and other outcomes that did not fit our definitions of SR, precluding their interpretation in the present case, such as Damaty (2020), Dolsen et al. (2019), and some of the analyses in Icenogle et al. (2019). As we will see later in the Results section, there were also several studies assessing the relationship between a pubertal variable and an outcome of interest, but whose results could not be interpreted in terms of effects of pubertal status.

Whenever there was a disagreement during the first stage of study selection, the study in question was automatically approved to the second selection stage and its full text was evaluated. Disagreements in the second stage were decided by consensus between the first and last authors of this study (Sabine Pompeia and Thiago F. A. França).

### 2.4 Data Extraction and Management

Relevant data from the included studies were extracted and inserted into spreadsheets by TFAF and checked by SP. We extracted the following data:

- Year of publication;
- Type of document;
- Goal of the study;
- Study design;
- Sample size;
- Participant information—age, sex, ethnicity, school grade, socioeconomic status (SES), country/region of origin;
- Pubertal measure;
- Outcome of interest;
- Approach to adjust results for age effects;
- Summary of findings and statistics of interest.

### 2.5 Criteria for Evaluating the Literature

To guide the critical evaluation of the literature, we adapted a set of relevant questions from the Critical Appraisal Skills Programme (CASP) checklist for cohort studies, section A (“are the results valid?”; available online at <https://casp‐uk.net/casp‐tools‐checklists/cohort‐study‐checklist/>), adding some criteria taken from the Joanna Briggs Institute Critical Appraisal Checklist for Analytical Cross Sectional Studies (available at <https://jbi.global/critical‐appraisal‐tools>). From this adaptation came the first version of the checklist, which is detailed in a document that can be found in the project's page at the OSF (https://doi.org/10.17605/OSF.IO/XFNUZ). The items of this initial version were refined and reorganized as we interacted with the studies themselves, leading to the final version described below.

The checklist was designed to ensure that the following points received systematic attention when interpreting the findings from the literature:

- Was the study design (longitudinal, cross‐sectional) appropriate to assess the role of pubertal status on SR/EF?
- What were the characteristics of the sample and could they affect the generalizability of the findings (external validity)?
- How informative were the pubertal measures to our goal of evaluating the effect of pubertal status on SR/EF development?
- How informative were the cognitive measures to our goal of evaluating the particular effect of pubertal status on SR/EF?
- Were there any unaccounted factors (such as differences in age, sex, SES, ethnicity, etc.) that could raise doubt about the conclusions (internal validity)?
- Related to the item above: (a) were the statistical analyses appropriate and did they match the conclusions regarding the impact of pubertal status on SR/EF?; and (b) what are the implications of the statistical assumptions to the significance of the reported statistics in the context of our review question?
- Regarding transparency, was the study preregistered and was the data available?

Of note, this checklist was not used to provide a score for studies, nor to classify or to include/exclude them in/from this review. Rather, it was meant as a tool to help make a more systematic critical evaluation of the findings of the literature as a whole.

It should also be kept in mind that the items above are not yes‐or‐no questions, and their answers go beyond simple assessments of a high or low risk of bias. When we reflected about the measures used, the characteristics of the studied populations, or the statistical models used to analyze data, there were multiple possible answers, and different answers have different potential implications for what each study can reveal regarding our research inquiry. For example, one study may assess puberty using the PDS, while the other measures blood levels of sex hormones. These different measures do not make one study necessarily better or more rigorous than the other, but the different measures provide different information and have different limitations, leading to distinct implications for interpreting the results. The same goes for studies using different measures of the same cognitive construct. Therefore, while it is standard practice to report quality/risk of bias assessments of each study as part of systematic reviews, such assessment of individual studies is not feasible in the present case. There is no clear and objective way to rate the quality and risk of bias of each study in terms of most criteria included in our questionnaire, even though those are the most important aspects determining the risk of bias in this literature. The only exceptions are the items related to transparency, involving the mention of preregistered protocols (which no study mentioned) and data availability statements (which only three studies reported), and those related to the adjustment of potentially relevant covariates in the analyses, which was rarely done in this literature. Information about these more objective aspects is provided for each study in Table 1. The remaining items, which varied more substantially in the literature, are not as straightforward to assess. Pros and cons of different study designs, sample characteristics, pubertal measures, cognitive/behavioral measures, and analytical approaches are often difficult to assess, and different approaches usually cannot be compared in a straightforward way. In the present case, it made more sense to discuss the varieties of methods used and how they impact the reliability of the literature as a whole, instead of assigning arbitrary scores for risk of bias to individual studies. The characteristics of each study can then be consulted in Table 1 and in our Supporting Information Tables, and linked with the common issues discussed in the main text. Accordingly, we assess and discuss the items in our checklist for the reviewed literature as a whole, and present our overall evaluation of each of the points listed above in the Results section, along with their implications to the interpretation of results from the reviewed literature.

## 3 Results

### 3.1 Search Results and Study Selection and Classification

Reference lists for the total search results, for studies approved in the first stage, and studies excluded by each criterion in the second stage of study selection, as well as for included studies, can be found in the project's page at the OSF (https://doi.org/10.17605/OSF.IO/XFNUZ).

**FIGURE 2:**
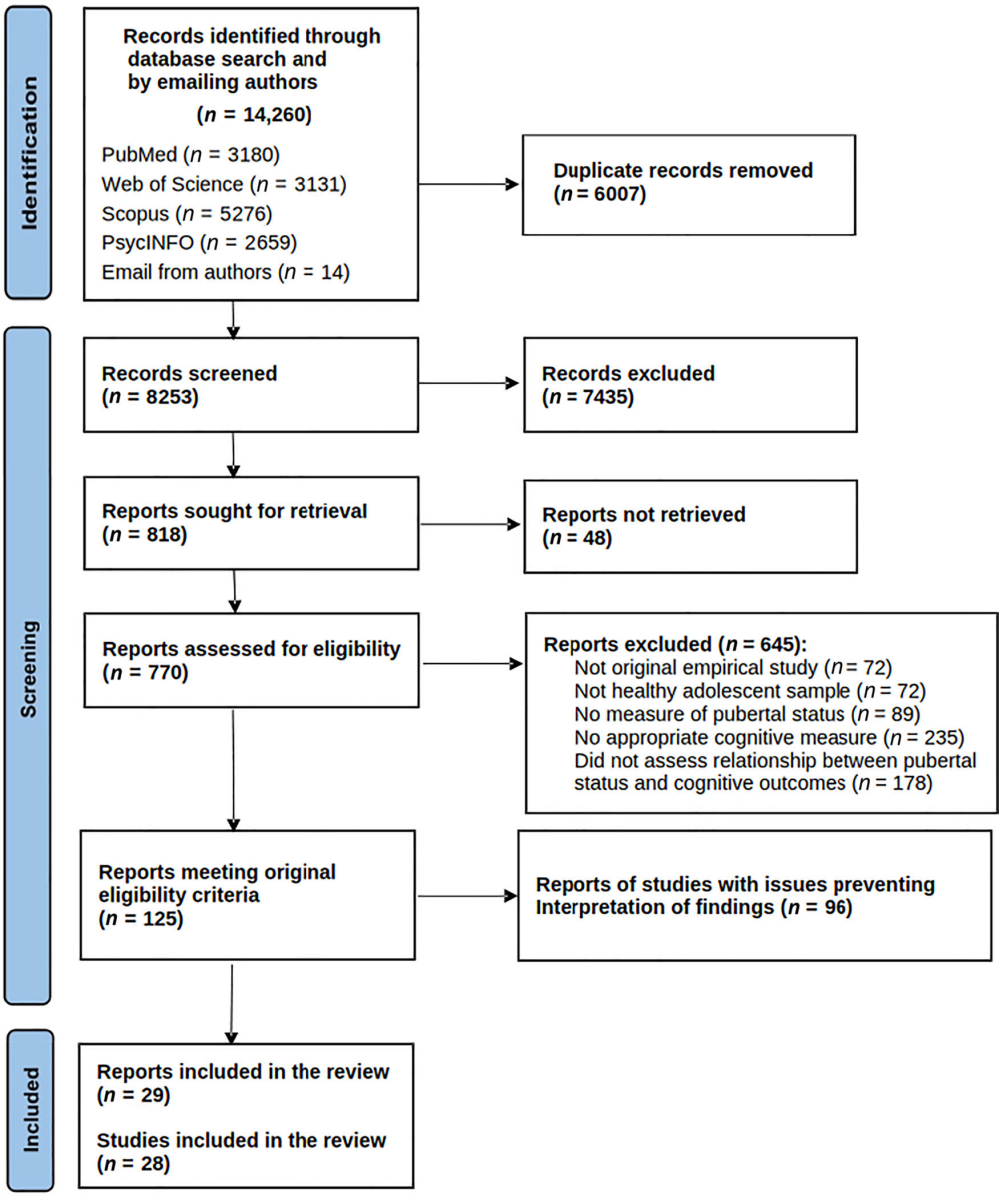
Flow diagram with the results of the study selection process. Of note, reports that presented “issues preventing interpretation of the findings” include studies that did not adjust for age effects, and/or adjusted for them in an insufficient or inadequate way, and/or used pubertal data to create variables that were not interpretable in terms of pubertal status, and/or studies that assessed pubertal status and self‐regulation/executive function outcomes at different ages. Many studies failed in more than one of these categories. For further discussion of this issue, see the Introduction and Discussion sections.

Our searches returned a total of 14,260 records. A flow diagram summarizing the results of the study selection can be found in Figure 2. After removing duplicates and applying the first five inclusion criteria to titles and abstracts, and then to the selected full texts, we were left with a total of 125 studies that reported results on the concomitant relationship between pubertal status and an outcome of interest. However, the results from most of those studies (*n* = 96) were not considered here because they did not allow inferences to be made about the direct association (or lack of) of pubertal status and SR/EF corrected/adjusted for age. Of note, this exclusion was not associated with the quality of the studies. It only meant that their goals did not exactly align with our review question.

These studies fail to address our research questions either because they:

1. Did not include chronological age in the statistical analyses nor controlled for it in the study design, or included age variables that lacked granularity. This category includes studies that did not use any method to account for age effects that may confound conclusions about the relationship between pubertal status and the cognitive/behavioral measures of interest. This was most common when studies were not primarily interested in the relationship between pubertal status and the SR measure, and only reported simple correlations between pubertal status and other study variables before proceeding to the studies’ main analyses. This category also includes studies that adjusted statistical analyses for school grade or for age in categories by the number of completed years. Such adjustments are insufficient because there is extensive evidence that pubertal status can change within a period of one full year (for a clear illustration, see data in Gracia‐Trabuenca et al. 2021) and that there are cognitive differences between older and younger individuals when age is counted in completed years, even within the same school grade (e.g., Peña 2020). Therefore, such adjustments can leave a significant part of age effects unadjusted for, especially in periods during which pubertal tempo tends to peak.
2. Adjusted or controlled for age in such a way that the scores used did not reflect the participants’ pubertal status per se. For example, several studies used measures commonly referred to as “(relative) pubertal timing” (Dorn and Biro 2011; Dorn et al. 2006; Mendle et al. 2019), usually obtained by regressing an indicator of pubertal status on the participant's age and then using the residuals as the independent variable for further analyses. This type of measure only reflects how advanced or delayed in sexual development each individual is compared to age and sex matched peers, so that participants of different pubertal status can receive the same relative pubertal timing score if they are of different ages, and the same participant may receive a different pubertal score if part of a different sample; and/or
3. Did not collect data on pubertal status and SR/EF performance in close proximity in time. This category was composed of a small number of studies, most of which used age of the timing of pubertal events/milestones (e.g., menarche, thelarche) as a measure of pubertal development and associated this to SR performance at later moments in time (i.e., their interest was mostly prospective effects of earlier or later absolute pubertal timing). Given the variations in the synchronicity and timing of different pubertal events, as well the significant variations in pubertal trajectories (tempo) between individuals (both of which can be seen when inspecting data from Biro et al. 2006, 2018; Legro et al. 2000; Pantsiotou et al. 2008; Vitalle et al. 2003), it is not possible to interpret the results of these studies in terms of the effects of pubertal status itself on SR, even if the analyses are adjusted for age at the time of cognitive assessment.

It should be noted that some studies fell in more than one of these categories, as they reported multiple results and analyses. In fact, even some of the studies included in the review have part of their results falling in some of these categories. We provide information about each of the studies excluded by these criteria, including clarifications about the precise issues that apply to each of them, in Table S1.

The net result was that only a minority of the initially selected reports (*n* = 29) were included in our review. Among these, there were two publications reporting the same results on the same sample: a paper (Herlitz et al. 2013) and a dissertation (Lovén 2012). Two other studies, Koch et al. (2020) and Mendle et al. (2020), seem to have used the same sample, but reported results from different measures. Ojha et al. (2022) also seem to have used part of the same sample from Ravindranath et al. (2022). Thus, we had a total of 29 reports, based on 28 studies, and involving 26 datasets, included in the review. The studies were published between 1985 and 2022 (although only two studies were published before 2001, and a total of 20 studies were published after 2010). These publications will be the focus of the rest of the review. They are summarized in Table 2 and further detailed in Table S2, available at the project's page at the OSF site (https://doi.org/10.17605/OSF.IO/XFNUZ).

**TABLE 2:** Summary of the characteristics of the studies selected for the review and their findings regarding the effects of pubertal status on self‐regulation/executive functions after adjusting for age.

Study id	Study design	Sample size	Participant demographics	Pubertal measure(s)	Outcome measure(s) of interest	Method to adjust for age effects	Summary of findings	Risk‐of‐bias issues (transparency and unaccounted demographic confounders)
Cardoos et al. 2017	Cross‐sectional.	63	**Age**: mean (SD) = 12.74 (1.09) years; range = 10–14 years. **Sex**: females only **Ethnicity**: 52.4% White; 22.2% Mixed race/ethnicity; 11.1% Black/African American; 7.9% Hispanic/Latino; 4.8% Asian; 1.6% Other **School grade**: not reported. **SES**: mean SES Community Ladder = 6.83 (0–10 scale) **Country/region of origin**: United States.	**Pubertal Development Scale** (PDS; Petersen et al. 1988). **Salivary testosterone, estradiol and DHEA,** analyzed with enzyme immunoassay kits.	**Airport Auction Task** (van den Bos 2008; 2013), adapted for the study.	Age was included as a covariate in the statistical models.	Both PDS and testosterone, but not estradiol and DHEA, had small‐to‐medium, positive effects (adjusted for age) on overbidding and negative effects on final earnings in the task.	Statistical analyses were not adjusted for ethnicity and school grade. No data availability statement. No information about a preregistered protocol.
Castagna and Crowley 2021	Cross‐sectional.	103	**Age**: mean (SD) = 14.49 (1.69) years. **Sex**: Both. 55 male, 48 female. **Ethnicity**: Caucasian (*n* = 79, 76.7%), African American (*n* = 9, 8.7%), Hispanic (*n* = 6, 5.8%), Asian (*n* = 6, 5.8%), other/unknown (*n* = 3, 2.9%). **School grade**: not reported. **SES**: not reported.	**Pubertal Development Scale** (PDS; Petersen et al. 1988).	**Flanker task**.	Age was included as a covariate in the statistical models.	Analyzing the parameters of two different decision models fitted to the task data, there was a small effect of the interaction between sex and PDS (adjusted for age), with a negative effect of PDS for females but not males on a parameter representing the amount of information that is considered for a decision. No other effects were found.	Statistical analyses were not adjusted for SES, ethnicity, and school grade. No data availability statement. No information about a preregistered protocol.
Chaku and Hoyt 2019	Longitudinal.	1099	**Age**: 9.5 years at baseline, 15.5 at last measurement. **Sex**: both. 51% female **Ethnicity**: 81.4% White, 11% Black and 7% of another race/ethnicity (black and other were collapsed in a single category). **School grade**: not reported. **SES**: youth had mothers with 14 years of education on average (i.e., some college), the average income‐to‐needs ratio was 4.37 (SD = 3.14), and 85% of sample lived in a two‐parent household (all SES variables assessed at age 9.5 only). **Country/region of origin**: United States.	**Tanner staging criteria** (Tanner 1962), assessed by experienced physician.	**Attention subscale of the Child Behavior Checklist** (CBCL; Achenbach and Edelbrock 1991), reported by the participants' mothers. **Self‐control subscale of the Social Skills Rating System** (SSRS; Gresham and Elliot 1990), reported by the participants' mothers. Note: both outcomes were assessed at ages 9.5, 10.5, 11.5 and 15.5.	Study used linear growth curve modeling to compute an intercept and slope from the time series of Tanner stages measured at the same ages (year and month) for all individuals.	Found a small, negative effect of pubertal status (adjusted for age) at baseline and social skills at baseline for girls. The effect remained when comparing data for the second wave instead of baseline. No other significant effects were found.	Statistical analyses were not adjusted for school grade. No information about a preregistered protocol.
Davison and Susman 2001	Longitudinal.	108	**Age (at baseline)**: for males: mean (SD) = 12.7 (1.32) years, range = 10–14 years; for females, mean (SD) = 11.99 (1.55) years, range = 9–14 years. **Sex**: both. 56 males and 52 females. **Ethnicity**: 97% Caucasian, 3% African‐American **School grade**: not reported. **SES**: participants were predominantly middle and upper–middle class. **Country/region of origin**: United States.	**Blood levels of testosterone and estradiol**, measured using radioimmunoassay. **Tanner stages** (Marshall and Tanner 1969, 1970; Tanner 1962), assessed by nurse practitioners.	**The spatial relations subscale of the Primary Mental Abilities test** (PMA; Thurstone 1962). **Block design subscale of the Wechsler Intelligence Scale for Children–Revised** (WISC‐R; Wechsler 1974).	Adjusted for age by adding it as a covariate in the regression analyses. However, age was not adjusted for in all relevant analyses, and so some of the analyses had to be excluded from the review.	There were positive, medium‐to‐large effects of testosterone (age adjusted) on block design and mental rotation scores for boys for data from waves 1 and 2. There was also a positive effect of estradiol on block design for data from wave 2 for males. Longitudinal analyses also found associations between linear trend scores for mental rotation and testosterone for males. For females, there was an effect of testosterone on mental rotation at wave 3. No other effects were found in the analyses that adjusted for age effects.	Statistical analyses were not adjusted for ethnicity, SES, and school grade. No information about a preregistered protocol. No data availability statement.
Deater‐Deckard et al. 2019	Cross‐sectional.	157	**Age: mean (SD) =** 14.07 (0.54) years; range = 13–15 years. **Sex: both**. 52% male **Ethnicity**: 82% White, 12% Black, 6% other. **School grade**: not reported. **SES**: 25% classified as “poor” (income‐to‐needs ratio—ITN < 1), 25% “near poor” (ITN < 2). The other 50% had ITN ≥ 2, with nearly half of these having ITN > 4. **Country/region of origin**: United States.	**Pubertal Development Scale** (PDS; Petersen et al. 1988).	**Multisource‐interference task** (MSIT; Bush et al. 2003).	Age was included as a covariate in the structural equation model.	No effects of PDS scores on task performance were found when adjusting for age.	Statistical analyses were not adjusted for sex, ethnicity, SES, and school grade. The study was not preregistered. No data availability statement.
Ellis 2002	Cross‐sectional.	165	**Age**: mean (SD) = 12.31 (1.58) years **Sex**: Both. 77 females; 71 males **Ethnicity**: presumed White by the authors. **School grade**: not reported. **SES**: not reported. **Country**/**region of origin**: United States.	**Body Changes Questionnaire**, parent‐ and self‐reported, a scale adapted from Carskadon and Acebo (1993), based on the PDS (Petersen et al. 1988).	**Early Adolescent Temperament Questionnaire—Revised** (EATQ‐R; Ellis and Rothbart 2002), self‐ and parental report, using the Effortful Control subscale of the further subdivided into subscales for attention, inhibitory control and activation control, (mostly) independent of socioemotional contexts.	Adjusted for age in the correlation analyses by calculating partial correlations.	For girls, there were significant, negative, small correlations between pubertal status adjusted for age and self‐reported attention, inhibitory and effortful control subscales (worse self‐control), and parent report or inhibitory and activation control (but not attention). No pubertal effects were found for males when adjusting for age.	Statistical analyses were not adjusted for SES and school grade. No information about a preregistered protocol. No data availability statement.
Gorday and Meyer 2018	Cross‐sectional.	99	**Age**: range = 8–14 years. **Sex**: females only. **Ethnicity**: not reported. **School grade**: not reported. **SES**: not reported. **Country/region of origin**: United States.	**Pubertal Development Scale** (PDS; Petersen et al. 1988), self‐ and parent‐reported. **Salivary concentrations of estradiol, progesterone, DHEA and testosterone,** assessed using enzyme immunoassay kits.	**Go/No‐go task**.	Adjusted for age in the correlation analyses by calculating partial correlations.	No effects of PDS or pubertal hormones on task performance were found when adjusting for age.	Statistical analyses were not adjusted for ethnicity, SES, and school grade. No information about a preregistered protocol. No data availability statement.
Graber et al. 2006	Cross‐sectional.	100	**Age**: mean (SD) = 12.13 (0.8) years; range = 10–14 years. **Sex**: females only. **Ethnicity**: White. **School grade**: 5th to 7th grade. **SES**: Most participants' families (96%) were in the two highest Hollingshead social classes. **Country/region of origin**: United States.	**Blood levels of DHEAS**, measured using radioimmunoassay kits. **Metrics**: The study seem to have used DHEAS concentration as a continuous variable.	**A 5‐item Attention scale was created based on items taken from the Youth Self‐report** (YSR; Achenbach and Edelbrock 1986).	Age was included as a covariate in the analyses.	No effects of DHEAS on questionnaire scores were found when adjusting for age.	Statistical analyses were not adjusted for SES. No information regarding a preregistered protocol. No data availability statement.
Herlitz et al. 2013/ Lovén 2012	Cross‐sectional.	187	**Age**: range = 12–14 years. **Sex**: both. 85 males and 102 females. **Ethnicity**: not reported. **School grade**: varied, but not reported in detail. **SES**: recruiting procedure was designed so that sample would be homogeneous with respect to socioeconomic background (upper middle class). **Country/region of origin**: Sweden.	**Tanner stages, self‐reported using text and schematic drawings developed for clinical use** (Hall and Pilström 1999, 1996). **Blood levels of estradiol and free‐testosterone**, measured using fluoroimmunoassay kits.	**Verbal fluency**. **Modified version of Vandenberg Mental Rotations Test** (Vandenberg 1971).	Adjusted for age in the correlation analyses by calculating partial correlations.	There was a positive, small‐to‐medium partial correlation (adjusted for age) between mental rotation and estradiol for boys. No other effects were found when adjusting for age.	Statistical analyses were not adjusted for school grade. No information about a preregistered protocol. No information on data availability.
Icenogle et al. 2017	Cross‐sectional.	3,23	**Age**: mean (SD) = 12.87 (2.36) years; range = 9–17 years. **Sex**: both. The proportions of male and female adolescents were nearly even within the whole sample (50.9% male, *n* = 1650; 49.1% female, *n* = 1709), within each country (range: 47.7%–53.5% female) and across ages. **Ethnicity**: varied, claimed to be representative of each country, but not reported. **School grade**: varied, not reported. **SES**: participants in each country came from households with similar levels of parental education, which averaged ‘some college’	**Pubertal Development Scale** (PDS; Petersen et al. 1988).	**Modified version of the Iowa Gambling Task** (IGT; Bechara et al. 1994).	Adjusted for age including it in the statistical models.	There was a medium‐sized, positive effect of PDS scores on the slope of the change in approach of the advantageous deck in the task (implying better performance). No other effects were found when adjusting for age.	Statistical analyses were not adjusted for school grade. No information regarding a preregistered protocol. No information on data availability.
			**Country/region of origin**: participants came from 11 countries: Guang‐Zhou and Shanghai, China (*N* = 321); Medellin, Colombia (*N* = 341); Nicosia, Cyprus (*N* = 233); Delhi, India (*N* = 240); Naples and Rome, Italy (*N* = 376); Amman and Zarqa, Jordan (*N* = 308); Kisumu, Kenya (*N* = 303); Manila, the Philippines (*N* = 309); several cities in the west of Sweden (*N* = 243); Chang Mai, Thailand (*N* = 321); and Durham and Winston‐Salem, the United States (*N* = 364).					
Koch et al. 2020	Longitudinal, but relevant analyses are cross‐sectional.	188	**Age**: mean (SD) = 11.75 (1.05) years; range = 9–14 years. **Sex**: females only. **Ethnicity**: European American (83%), Southeast Asian (5.24%), East Asian/Pacific Islander (3%), American Indian/Native (2.25%), African American (1.87%), Hispanic/Latino (1.12%) and bi‐racial or another race (3.37%). **School grade**: Varied. **SES**: 87% of parents who reported their education (*N* = 75) holding a bachelor's degree or higher. But most parents did not report schooling indicating reporting bias. **Country/region of origin**: United States. Of note, this seems to be the sample studied in Mendle et al. 2020.	**Pubertal Development Scale** (PDS; Petersen et al. 1988; Carskadon and Acebo 1993).	**Ruminative Response Scale of the Children's Response Styles Questionnaire** (Abela et al. 2002; Abela et al. 2007).	Age was included as a covariate in the relevant structural equation models.	There was a small, positive effect of pubertal status on rumination scores, meaning increase of rumination.	Statistical analyses were not adjusted for ethnicity, SES, and school grade. No data availability statement. No information regarding a preregistered protocol.
Kovács et al. 2022	Cross‐sectional.	117	**Age**: range = 11–15 years old. **Sex**: females only. **Ethnicity**: not reported. **School grade**: not reported. **SES**: 90.6% of mothers and 88.0% of fathers has a University degree. **Country/region of origin**: Hungary.	**Skeletal maturity** (bone age), assessed with an ultrasonic device.	**Subtests from the Wechsler Intelligence Scale for Children IV** (Wechsler 2003): Block Design, Visual Puzzles, and Matrix Reasoning were used to compose a Perceptual Reasoning Index; Digit Span and Letter‐Number Sequencing were used to compose a Working Memory Index.	Adjusted for age in the correlation analyses by calculating partial correlations.	The results showed a significant, small‐to‐moderate, positive correlation of bone age with the working memory index. No statistically significant correlations were observed with the perceptual reasoning index.	Relevant statistical analyses were not adjusted for ethnicity, SES, and school grade. No information regarding a preregistered protocol.
Kretsch and Harden 2014	Cross‐sectional.	58	**Age**: mean (SD) = 13.6 (1.67) years; range = 11–16 years. **Sex**: both. Male and female (50%). **Ethnicity**: African‐American (63.9%), Hispanic (19.7%) and Caucasian (presumably 16.4%). **School grade**: not reported. **SES**: 76% of participants reported receiving free or reduced‐price lunch at their school, indicative of low income. **Country/region of origin**: United States.	**Modified version of the Pubertal Development Scale** (PDS; Petersen et al. 1988), with separate scores for items related to gonadal hormones and items related to adrenal hormones.	**The Stoplight Game** (Chein et al. 2011; Gardner and Steinberg 2005; Steinberg et al. 2008) played alone or observed by peers.	Age was included as a covariate in the statistical model.	There was an effect of pubertal status based on gonadal score, with a positive effect on the number of risky decisions (i.e., higher risk‐taking) in the task irrespective of playing alone or with peers. There was a significant effect of equal direction and similar magnitude for adrenal scores on latency to wait only in the peer condition.	Statistical analyses were not adjusted for school grade. No data availability statement. ‍No information regarding a preregistered protocol.
Laube et al. 2017	Cross‐sectional.	72	**Age**: mean (SD) = 12.34 (1.17) years; range = 11–14 years. **Sex**: males only. **Ethnicity**: not reported. **School grade**: not reported. **SES**: not reported. **Country/region of origin**: United States.	**Salivary testosterone**, measured by unspecified method. **Pubertal Development Scale** (PDS; Petersen et al. 1988).	**An intertemporal choice task** (McClure et al. 2004).	Age was included as a covariate in the statistical model.	There was a small‐to‐medium, positive effect of testosterone on the proportion of smaller and sooner choices (higher impulsiveness) in the task. No other effects were found when adjusting for age.	Statistical analyses were not adjusted for ethnicity, SES, and school grade. No information regarding a preregistered protocol. No data availability statement.
Laube et al. 2020	Cross‐sectional.	70	**Age**: mean (SD) = 12.56 (1.64) years; range = 11–15 years. **Sex**: males only. **Ethnicity**: not reported. **School grade**: not reported. **SES**: not reported. **Country/region of origin**: Germany.	**Salivary testosterone**, measured by ELISA.	**An intertemporal choice task** (Rodriguez et al. 2015).	Age was included as a covariate in the statistical model.	There was a small‐to‐medium, positive effect of testosterone on the bias for smaller and sooner choices in the task (higher impulsiveness). No other effects were found when adjusting for age.	Statistical analyses were not adjusted for ethnicity, SES, and school grade. No information regarding a preregistered protocol. No data availability statement.
Lee and Rasmussen 2022	Cross‐sectional.	28	**Age**: mean (SD) = 11.21 (0.44) years; range = 9–16 years. **Sex**: both. 8 females, 20 males. **Ethnicity**: 25 (89.3%) White, 2 (7.1%) Hispanic/Latino, 1 (3.6%) Asian. **School grade**: not reported. **SES**: not reported. **Country/region of origin**: United States.	**Tanner staging criteria** (Marshall and Tanner 1969, 1970), assessed by a physician.	**Delay discounting** was assessed with two tasks: **Food Choice Questionnaire** (FCQ; Hendrickson et al. 2015; Hendrickson and Rasmussen 2017). **Monetary Choice Questionnaire** (MCQ; Kirby and Marakovic 1996; Kirby et al. 1999).	Age was included as a covariate in the statistical models.	No effects of pubertal status on discounting variables were found when adjusting for age.	Statistical analyses were not adjusted for ethnicity, SES, and school grade. No data availability statement. No information regarding a preregistered protocol.
Mendle et al. 2020	Cross‐sectional.	228	**Age**: mean (SD) = 11.75 (1.05) years; range = 9–14 years. **Sex**: females only. **Ethnicity**: European American (79.2%), Southeast Asian (2.21%), East Asian/Pacific Islander (2.21%), American Indian/Native (3.54%), African American (2.21%), Hispanic/Latino (3.98%), and biracial/another race (6.64%). **School grade**: varied. **SES**: 87% of parents who reported their education (*N* = 75) holding a bachelor's degree or higher. But most parents did not report and there is evidence of reporting bias. **Country/region of origin**: United States. Note: this seems to be the sample studied in Koch et al. (2020).	**Pubertal Development Scale** (PDS; Petersen et al. 1988; Carskadon and Acebo 1993).	**Negative Urgency** subscale from the UPPS‐P Impulsive Behavior Scale for Children (Zapolski et al. 2010). Ruminative Response Scale of the Children's Response Styles Questionnaire (Abela et al. 2002; Abela et al. 2007).	Age was included as a covariate in the relevant structural equation models.	There was a small, positive effect of pubertal status on rumination scores when adjusting for age. There were no direct effects of pubertal status on negative urgency when adjusting for age.	Statistical analyses were not adjusted for school grade. No information regarding a preregistered protocol. No data availability statement.
Ng‐Knight et al. 2016	Longitudinal.	750 at wave 1, 1712 at wave 2 and 1653 at wave 3.	**Age (at baseline)**: mean (SD) = 11.25 (0.29) years. **Sex**: both. 54% female. **Ethnicity**: 40% from “minority ethnic groups” **School grade**: beginning secondary school at baseline. **SES**: 16% had “indicators of socioeconomic deprivation” **Country/region of origin**: United Kingdom.	**Pubertal Development Scale** (PDS; Petersen et al. 1988), self‐reported. PDS scores were obtained in each of the three waves.	**13‐item Brief Self‐Control Scale** (BSCS; Tangney et al. 2004), self‐report. Self‐control was assessed in each of the three waves of the study.	Adjusted for age by including it as a covariate in the structural equation models.	There was a small, negative effect of pubertal status at baseline on self‐control at baseline. No other effects were found when adjusting for age.	Statistical analyses were not adjusted for ethnicity and school grade. No information regarding a preregistered protocol. No data availability statement.
Ojha et al. 2022	Longitudinal	106	**Age**: range = 10–18 years. **Sex**: 50 females, 56 males **Ethnicity**: not reported. **School grade**: not reported. **SES**: not reported. Country/region of origin: Pittsburgh, PA, United States. Of note, the sample in this study was also analyzed in Ravindranath et al. 2022.	**Pubertal Development Scale** (PDS; Petersen et al. 1988).	**An antisaccade task** (Ordaz et al. 2018; Ravindranath et al. 2022).	Age was included as a covariate in the statistical models.	The results showed no significant association between antisaccade performance (i.e., proportion of correct trials) and pubertal maturation after adjusting for age.	‍Statistical analyses were not adjusted for ethnicity, SES, and school grade. No information regarding a preregistered protocol. No data availability statement.
Olson et al. 2009	Cross‐sectional.	79	**Age**: mean (SD) = 16.44 (4.10) years; range = 9–23 years. **Sex**: both (53.2% female). **Ethnicity**: of 72 participants: Caucasian (68), African American (1), Hispanic (3), Asian/Pacific Islander (2), other (including multiracial) (4), and not reported (1). **School grade**: not reported. **SES**: varied; income mean (SD) = 91,493 (71,292). **Country/region of origin**: United States.	**Pubertal Development Scale** (PDS; Petersen et al. 1988).	**A delay‐discounting task** (Richards et al. 1999).	Age was included as a covariate in the statistical models.	There were no effects of PDS scores on discounting parameter when adjusting for age.	Statistical analyses were not adjusted for sex, ethnicity, SES, and school grade. No information regarding a preregistered protocol. No data availability statement.
Ordaz et al. 2018	Cross‐sectional.	78	**Age**: range = 11–13 years for females, 12–14 years for males. **Sex**: both. 34 females, 44 males. **Ethnicity**: 78% White, nonHispanic, 14% Black, nonHispanic, and 8% multiracial. **School grade**: not reported. **SES**: not reported. **Country/region of origin**: United States.	**Tanner staging criteria** (Marshall and Tanner 1968), assessed by a nurse. **Blood levels of testosterone (for boys and girls) and estradiol (only for girls)**, using radioimmunoassay kits.	**An antisaccade task** (Hartmann et al. 2013).	Age was included as a covariate in the statistical models.	There were no effects of pubertal status or hormone levels on accuracy in the task when adjusting for age.	Statistical analyses were not adjusted for ethnicity, SES, and school grade. No information regarding a preregistered protocol. No data availability statement.
Ravindranath et al. 2022	Longitudinal	105, who completed 227 visits	**Age**: range = 8.00–19.3 years old. **Sex**: Both. 53 females, 52 males. **Ethnicity**: not reported. **School grade**: not reported. **SES**: not reported. **Country/region of origin**: Pittsburgh, PA, United States. **Of note, part of this sample was also analyzed in** **Ojha et al**. 2022.	**Pubertal Development Scale** (PDS; Petersen et al. 1988). **Tanner staging criteria assessed via a self‐report scale with line drawings of Tanner stages** (Morris and Udry 1980).	**An antisaccade task** (Hwang et al. 2010; Ordaz et al. 2018; Velanova et al. 2009, 2008).	Age was included as a covariate in the statistical models.	There were no significant effects of pubertal status on task performance after adjusting for age.	Statistical analyses were not adjusted for ethnicity, SES, and school grade. No information regarding a preregistered protocol.
Steinberg et al. 2008	Cross‐sectional.	417	**Age**: range = 10–16 years. **Sex**: both. 231 males and 186 females. **Ethnicity**: 30% African Americans, 15% Asians, 21% Latino(a)s, 24% Whites, and 10% others in the whole sample (ages 10–30). Not reported for the subsample of interest. **School grade**: not reported. **SES**: “predominantly working and middle class.” Assessed via parent's education. **Country/region of origin**: United States.	**Pubertal Development Scale** (PDS; Petersen et al. 1988).	**The Stoplight Game** (Gardner and Steinberg 2005).	Age was included as a covariate in the statistical models.	Pubertal status had a positive effect on the number of intersections crossed successfully in the task (better performance). No other effects were found when adjusting for age.	Statistical analyses were not adjusted for ethnicity and school grade. No information regarding a preregistered protocol. No data availability information.
Sullivan et al. 2016	Cross‐sectional.	692	**Age**: range = 12.0–21.9 years. **Sex**: both. 344 male and 348 female. **Ethnicity**: most participants were Caucasian (486), African‐American (87) and Asian (52). **School grade**: Not reported. **SES**: years of parental education, mean (SD) ∼16.8 (2.5). **Country/region of origin**: United States.	**Pubertal Development Scale** (PDS; Petersen et al. 1988).	University of Pennsylvania Web‐Based Computerized Neurocognitive Battery (WebCNP; Gur et al. 2012; Gur et al. 2010) including composite scores for speed and accuracy in abstraction (including conditional exclusion, matrix and logical reasoning), attention (including various measures of a continuous performance task) and working memory (various measures of a visual N‐back task).	States that analyses were performed to evaluate effects of pubertal development independent of age, but no details are given.	No independent contributions of age versus PDS to performance were found.	Statistical analyses were not adjusted for school grade. No information regarding a preregistered protocol. No data availability statement.
Vannucci et al. 2014	Cross‐sectional.	468 or 122, depending on the analysis.	**Age**: mean (SD) = 14.01 (2.53) years, rage = 8–17 years. **Sex**: both. 53.4% female. **Ethnicity**: 58.1% non‐Hispanic White, 33.3% non‐Hispanic Black or African American, 5.9% Hispanic/Latino, 2.3% Asian Origin, and 1.4% Multiple Races. **School grade**: not reported. **SES**: not reported. **Country/region of origin**: United States.	**Tanner breast staging criteria for girls and Prader testicular volume standards for boys** (Marshall and Tanner 1969, 1970; Tanner 1981), assessed by endocrinologist or trained nurse.	**Objective and subjective binge episodes (OBE and SBE, respectively) with loss of control, assessed with the Eating Disorder Examination (EDE), versions 12.0D and C.2** (Fairburn and Cooper 1993; Bryant‐Waugh et al. 1996).	Age was included as a covariate in the statistical models.	There were no effects of PDS on outcomes of interest when adjusting for age.	Statistical analyses were not adjusted for SES and school grade. No information regarding a preregistered protocol for the analyses. No data availability statement.
Vetter et al. 2013	Cross‐sectional.	60	**Age**: mean (SD) = 13.86 (0.92) years; range = 12–15 years. **Sex**: both (23.3% male). **Ethnicity**: not reported. **School grade**: 7th and 8th grades. **SES**: not reported. **Country/region of origin**: Germany.	**Pubertal Development Scale** (PDS; Petersen et al. 1988), self‐reported German version (Watzlawik 2009).	**Story Comprehension test** (Channon and Crawford 2000), a theory of mind task translated into German.	Age was included as a covariate in the statistical models.	There were no effects of PDS on outcomes of interest when adjusting for age.	Statistical analyses were apparently not adjusted for sex, ethnicity, SES, and school grade. No information regarding a preregistered protocol. No data availability statement.
Waber et al. 1985	Longitudinal, but relevant analyses are cross‐sectional.	145	**Age**: mean (range) = 127.41 (120–134) months for females and 153.4 (145–162) months males. **Sex**: both. 78 females and 67 males. **Ethnicity**: White **School grade**: 5th grade (females) and 7th grade (males). **SES**: fathers' occupations were rated according to the Hollingshead system. Of the 90% of the girls for whom parental occupation was known, 93% were classified as upper to upper‐middle class. Similarly, for the boys, of the 91% for whom parental occupation was known, 89% were classified as upper to upper‐middle class. **Country/region of origin**: United States.	**Tanner staging criteria** (Tanner 1962), assessed by a physician.	Several cognitive tests: **Stroop Color‐Word Interference Test; Wechsler Intelligence Scale for Children‐Revised (WISC‐R) Coding Subtest; WISC‐R Block Design; Primary Mental Abilities (PMA) Spatial Test; PMA Word Fluency Test**.	Age was included as a covariate in the statistical models.	There were negative, statistically significant correlations (adjusted for age) between pubertal status and scores adjusted for age for coding and block design for females. However, the magnitude of the adjusted correlations was not reported and these correlations could only be found when separately analyzing data for participants from one of the two study towns.	No information regarding a preregistered protocol. No data availability statement.
Warren and Brooks‐Gunn 1989	Cross‐sectional.	100	**Age**: mean = 12.1 years; range = 10.6–13.3 years. **Sex**: females only. **Ethnicity**: only White. **School grade**: 5th‐7th grade. **SES**: middle to upper–middle class. Ninety‐six percent of the families were in the two highest of the five Hollingshead social classes. **Country/region of origin**: United States.	**Tanner staging criteria** (Marshall and Tanner 1969), assessed by a nurse or physician. Blood levels of **LH**, **FSH**, **prolactin**, **estradiol**, **testosterone**, and **DHEAS**, measured by radioimmunoassay.	**Impulse Control subscale of the Self Image Questionnaire for Young Adolescents** (SIQYA; Petersen et al. 1984).	Age was included as a covariate in the statistical models.	There was a significant quadratic relationship between impulse control scores with stages based on hormonal concentrations. Impulse control decreased between hormonal stages I and II, and increased between II–III and III–IV. No other effects were found when adjusting for age.	Statistical analyses were not adjusted for SES and school grade. No preregistered protocol. No data availability statement.

### 3.2 Characteristics of Included Studies

#### 3.2.1 Design

Only five of the 28 studies reported longitudinal analyses involving measures of interest to this review (Davison and Susman 2001; Ng‐Knight et al. 2016; Chaku and Hoyt 2019; Ojha et al. 2022; Ravindranath et al. 2022). Another two studies were also longitudinal (Koch et al. 2020; Waber et al. 1985), but all of the reported analyses of interest were cross‐sectional comparisons. All other studies used cross‐sectional designs. We stress that most of the studies were on relatively small samples, with only seven studies having samples with more than 200 participants (see details in Table 2 and further information in Table S2).

#### 3.2.2 Sample Characteristics

All but six of the 28 reviewed studies involved participants from the United States. The exceptions were two studies with participants from Germany (Vetter et al. 2013; Laube et al. 2020), one with participants from the United Kingdom (Ng‐Knight et al. 2016), one each with participants from Sweden (Herlitz et al. 2013/Lovén 2012) and Hungary (Kovács et al. 2022), and another involving participants from multiple countries (Icenogle et al. 2017). Nineteen of the 28 studies involved samples with both male and female participants, the remainder having tested only male (*n* = 2) or only female participants (*n* = 7).

As can be seen in Table 2, all studies reported information on the participants’ ages, either as age ranges or mean and standard deviations, and sometimes both. Based on these data, we can see that most studies focused on early‐ and midadolescents (which makes sense as this is when the pubertal transition takes place) and only four studies had samples well into late adolescence/early adulthood (Olson et al. 2009; Sullivan et al. 2016; Ojha et al. 2022; Ravindranath et al. 2022).

Across studies that reported participants’ ethnicity/race (*n* = 20), the vast majority of participants were classified as being White. Only a few studies had a majority of non‐White participants (Steinberg et al. 2008; Kretsch and Harden 2014; and likely Icenogle et al. 2017, a cross‐cultural study which claims its samples reflected the dominant ethnicity of their respective countries).

As for participant's SES, this variable was not reported in almost half of the studies, as can be seen in Table 2. Among those that did provide data on SES (*n* = 15), there was a mix of metrics that were often difficult to compare. Nonetheless, apart from the studies by Deater‐Deckard et al. (2019) and Kretsch and Harden (2014), most studies reporting SES seem to have included a majority of participants from middle‐ to upper‐class families.

#### 3.2.3 Measures of Pubertal Status

The most common measure of pubertal status used in the eligible studies was the PDS (Petersen et al. 1988; Carskadon and Acebo 1993) or adapted versions, which were used by a total of 16 studies. This is a self‐ or parent‐reported questionnaire with items that enquire about perceived adrenal and gonadal pubertal changes in skin, height, and armpit hair (for both sexes), breast growth and occurrence of menarche (for females), and facial hair growth and voice changes (for males). Each item is scored on a 4‐point scale (1: has not yet started; 4: seems complete), except for the item in respect of menarche, with either 1 or 4 points scored for not having or having experienced menarche, respectively. Total scores are usually the average or the sum of the scores of the five questions.

Eight studies measured pubertal status using Tanner stages (Marshall and Tanner 1969, 1970), mostly assessed by a nurse or physician. This method assigns adolescents to one of five possible Tanner stages (from pre‐ to postpubertal) based on pubic hair in both sexes and breast development in females or genital development in males.

Additionally, there were nine studies that measured blood or salivary concentrations of sex hormones. Seven of these used both hormonal assays and measures of physical development (either the PDS or Tanner stages). Finally, there was a single study (Kovács et al. 2022) that used an ultrasound device to estimate bone age from acoustic parameters of participants’ left wrist.

#### 3.2.4 Measures of SR/EF

Most studies included only a single measure to assess cognitive abilities related to our operational definition of hot and cool SR/EF. As expected, given the broadness of the SR/EF construct, these measures varied widely among studies and included a set of highly heterogeneous laboratory tasks and questionnaires. Various studies assessed some traditional (nonemotional/social) EF tasks, which are commonly associated with control of attention, but each type of ability was generally represented by only one or two tasks (e.g., tasks for working memory updating, continuous performance/sustained attention tasks, inhibition or inhibitory control, verbal fluency, and mental rotation).

As can be seen in Table 2 (and in more detail in Table S2), the most commonly investigated construct was “inhibition” or “inhibitory control,” loosely defined as the capacity to inhibit automatic, habitual, and inappropriate responses. The case of inhibition provides a stark illustration of the heterogeneity of the instruments used by different studies, as there was significant variation in the instruments supposedly measuring this very construct. For example, among the laboratory tasks used to measure inhibition there were a range of tasks, including the Flanker task, the antisaccade task, the Stroop test, and the Go/No‐go test, which reflect SR/EF skills in nonemotional/social conditions (cool).

There were also laboratory tasks assessing decision‐making under socioemotionally salient conditions. Again, heterogeneity of measures was high, and this construct was assessed using different intertemporal choice paradigms (delay discounting tasks with rewards) and decision‐making under risk/uncertainty (e.g., the Iowa Gambling Task, the Airport Auction Task, and the Stoplight Game).

Heterogeneity was also observed in the assessment of SR/EF abilities using questionnaires that measure, according to the authors, constructs such as “inhibition,” “attention,” “self‐control,” “emotion regulation,” and so forth, which are terms that can only be very loosely defined based on their use across studies.

A careful analysis of the content of the questions or statements in the instruments used led us to classify them as (mainly) socioemotionally independent (cool) or not (hot). Hence, “cooler” measures about SR/EF difficulties in adolescents’ lives included scales such as the Brief Self‐Control Scale (BSCS), and the Effortful Control subscale of the Early Adolescent Temperament Questionnaire—Revised (EATQ‐R). As for daily difficulties associated with SR/EF in socioemotionally salient conditions, a greater variety of measures were used, including different scales (Social Skills Rating System [SSRS], Ruminative Response Scale of the Children's Response Styles Questionnaire, the Negative Urgency subscale from the UPPS‐P, the Impulsive Behavior Scale for Children—all self‐reported, except for the SSRS, which was parent‐reported) and a single task that was used to explore social cognition/theory of mind (a story comprehension task).

#### 3.2.5 Statistical Analyses and the Treatment of Age Effects and Other Putative Confounders’

The statistical methods and approaches employed in the reviewed studies were widely diverse and each study had their own, including: partial correlations, analyses of variance/covariance (ANOVAs/ANCOVAs), hierarchical regression models, and structural equation models (information about the analyses of each study are provided in Supporting Information Table 2). This diversity was also visible in the way age effects were accounted for. Except for Chaku and Hoyt (2019), who tested participants at the same ages prospectively, that is, controlled for age in the study design, the remaining studies adjusted for age effects in the statistical analyses. This was done by adding age as a covariate in the statistical models (e.g., ANCOVAs, regression models, or structural equation models). Information on the methods used in each study can be found in Table 2 and, in greater detail, in Table S2. As for other possible confounders, most studies analyzed in this review did not adjust the statistical analyses for SES or ethnicity/race, so these effects will only be mentioned when these studies are described below.

#### 3.2.6 Transparency: Preregistration and Data Availability

Only three of the 28 studies (Chaku and Hoyt 2019; Kovács et al. 2022; Ravindranath et al. 2022) included a data availability statement. No study mentioned preregistered protocols.

### 3.3 Summary Description of Studies’ Findings

Overall, 11 of the 28 eligible studies found no statistically significant effects of pubertal status on outcomes measures of SR/EF after controlling/adjusting for age (see Tables 2 and S2 for more details). As for the remaining studies, there were usually a few significant effects among an equal or greater number of nonsignificant results. Overall, the statistically significant results reported were effects of small‐to‐medium size, though some studies did not report effect sizes, and often the precision of reported effect sizes could not be properly assessed based on the reported statistics (e.g., estimates reported without confidence intervals; see Table S2 for more details on this point). As can be seen below and in Tables 2 and S2, both positive and negative pubertal effects on SR/EF abilities were reported. In the following section, we briefly describe the results reported in this literature.

Of note, while all studies measured aspects of SR/EF, not all instruments are directly comparable and a classification of the results was necessary for a coherent analysis/synthesis. This classification, however, was not straightforward because: (1) there is no established consensual list of the types of cognitive abilities under the umbrella‐term of SR/EF; (2) studies used distinct terminologies to name the abilities they assessed, at times calling the same construct by different names, or different abilities by the same name (jingle–jangle fallacies); and (3) a variety of SR/EF abilities were sampled and, at the same time, many possible SR/EF domains were not represented in the selected studies.

Based on a careful analysis of the description of the tasks and questionnaires reported in this literature, and taking into account our working definition of SR/EF, we divided the studies in three main recognizable categories as follows: (A) decision‐making laboratory tasks, which mostly assess hot skills; (B) SR/EF in real‐life scenarios assessed with questionnaires that inquire about both hot and cool abilities; and (C) classic laboratory tasks associated with cool SR/EF abilities, such as EFs, working memory, and attention. For each of these three categories, we initially summarized the findings and then detailed the results, as described next.

- Decision‐making in laboratory tasks

The six studies in this category involved decision‐making tasks of two main subtypes: decision‐making under risk/uncertainty and intertemporal choice tasks (involving choices with sure‐win options), both of which involve rewards. All of these measures are usually regarded as assessing hot SR/EF abilities.

- Decision‐making in laboratory tasks under risks/uncertainty

Four studies assessed decision‐making involving risks and gains: Steinberg et al. (2008), Kretsch and Harden (2014), Cardoos et al. (2017), and Icenogle et al. (2019). The results reported, however, were inconsistent. While the studies by Kretsch and Harden (2014) and Cardoos et al. (2017) found negative effects of pubertal status on task performance, the studies by Steinberg et al. (2008) and Icenogle et al. (2019) reported positive effects, as detailed below.

Cardoos et al. (2017) involved a sample of female adolescents and found a negative effect of PDS scores and testosterone concentrations on performance in the Airport Auction Task, which involves taking financial risks with virtual money to win the game and gain social status (having their photo shown to other players). Higher PDS scores and testosterone levels were associated with higher overbidding (willingness to wager above a neutral risk bid) and lower final earnings after adjusting for age, SES, and vocabulary.

In contrast, Icenogle et al. (2017) studied adolescents from both sexes and reported a positive effect of pubertal status on some of the analyzed aspects of adolescent performance in the Iowa Gambling Task. This task involves maximizing winnings by choosing cards from different decks that unpredictably differ in the balance between reward and penalty cards; however, some decks lead to higher gains but also higher losses, leading to smaller final payouts so must be avoided despite providing much higher trail by trial gains. Performance was adjusted for age, sex, SES, and intelligence (measured by the matrix reasoning subtest of the Wechsler Abbreviated Scale of Intelligence).

Steinberg et al. (2008) and Kretsch and Harden (2014) both studied samples including male and female adolescents and investigated the relationship between pubertal status (PDS scores) and behavior in a simulated driving task, the Stoplight Game, using different scores. This task involves uncertain outcomes (timing of traffic lights at intersections and probability of crashes) under which players must decide whether to avoid risks (brake to stop at a yellow light, which results in losing time/points until the traffic light returns to green), take a small risk with a low pay off or opt to take a high risk with high payoff or loss (e.g., going through a yellow light, which can save time/gain points but can lead to a crash/loss of points).

Participants in Steinberg et al. (2008) were told they would receive monetary rewards depending on their performance, while Kretsch and Harden (2014) had them carry out the task alone and watched by peers (no apparent financial reward for their performance, although peers’ presence can serve as a reinforcer). Steinberg et al. (2008) found that pubertal status was not related to safe stopping, risky driving, or crashing, although higher pubertal status was associated with better performance (a higher number of successful intersection crossings) after adjusting for intelligence (assessed using the vocabulary and matrix reasoning subtests of the Wechsler Abbreviated Scale of Intelligence), SES, and age.

Kretsch and Harden (2014), on the other hand, found the opposite pubertal effect: more pubertally mature individuals (based on a gonadal score derived from a subset of items from the PDS) made a greater percentage of risky choices irrespective of carrying out the task alone or watched by peers (similar analyses were carried out with the adrenal scores, but did not show significant results). Their model was controlled for age, phonological working memory (assessed using a digit span test), sex, and ethnicity.

- Decision‐making involving choices between sure‐win options

Four studies reported pubertal effects on delay discounting measures. All four of them seem to have employed hypothetical rewards and delays, meaning participants were expected to imagine their preferences if given a choice between waiting longer to get a bigger virtual reward or receiving a smaller virtual reward sooner. Two of these studies showed fairly consistent negative pubertal status effects (Laube et al. 2020, 2017), with more pubertally advanced participants (based on salivary testosterone levels or PDS scores) preferring immediate, smaller rewards. However, the two other studies found no pubertal status effects: Olson et al. (2009) and Lee and Rasmussen (2022).

Laube et al. (2017) studied a sample of male adolescents. They built statistical models relating the proportion of smaller sooner choices in an intertemporal choice task to testosterone levels, age, and different task conditions (smaller immediate vs. larger later reward; or two delayed choices, where the larger delay was associated with higher reward). They found an interaction of task condition and testosterone, with individuals with higher testosterone levels preferring smaller virtual immediate reward choices. Laube et al. (2017) also fitted a model of temporal‐discounting behavior with two key‐parameters: the discount rate, *k*, and the parameter, *s*, which represented the relative sensitivity to more immediate versus later rewards. The only significant result found was a positive, age‐adjusted correlation between the parameter *s* and testosterone concentrations.

Laube et al. (2020) used the same measures as the one employed in the study by Laube et al. (2017), and fitted another delay discounting model to data from another set of male adolescents. They tried to predict the level of bias, *s*, for the smaller sooner options and the parameter *k* based on both testosterone concentrations and age. The only effect observed was that adolescents who had higher levels of testosterone also showed an increased response bias to the smaller sooner option.

Contrary to the two studies above, Olson et al. (2009), and Lee and Rasmussen (2022), found no pubertal status effects on delay discounting measures controlling for age. Lee and Rasmussen (2022) applied two variations of delay discounting questionnaires to adolescents of both sexes, a traditional one with virtual monetary rewards, and a variation with virtual food rewards, and used Tanner stages controlling additionally for body mass index/percentage of body fat. Olson et al. (2009) used PDS scores and a similar monetary delay discounting task as that of Lee and Rasmussen, with one key difference: after the task, one of the trials was randomly selected and participants received a real cash reward related to their choice in that trial. None of these studies reported associations between pubertal status and performance.

- SR/EF difficulties in real‐life scenarios (assessed with questionnaires)

The nine studies that assessed SR/EF in real‐life employed questionnaires/scales which could be further separated into: (i) questionnaires involving SR/EF difficulties mostly under socioemotional scenarios (hot), which were almost all self‐rated; and (ii) questionnaires that enquired about these difficulties in mostly socioemotional neutral conditions (cool).

- SR/EF in socioemotional contexts (hot)

Overall, of the seven studies that assessed this, small negative effects of pubertal status were found on self‐reported self‐control difficulties using different questionnaires/scales that inquire about adolescents’ behavior in socioemotional salient (hot) conditions in their daily lives (Warren and Brooks‐Gunn 1989; Vetter et al. 2013; Ng‐Knight et al. 2016; Chaku and Hoyt 2019; Koch et al. 2020; Mendle et al. 2020). These effects, however, were only consistently present in females in early puberty (Warren and Brooks‐Gunn 1989; Ng‐Knight et al. 2016; Chaku and Hoyt 2019), who displayed more difficulties in SR/EF. However, results from Vannucci et al. (2014) and Vetter et al. (2013), as well as part of the results from Mendle et al. (2020), showed no pubertal effects on three similar scales.

Chaku and Hoyt (2019) conducted a 7‐year‐long longitudinal study assessing participants’ Tanner stages and self‐control (using a subscale of the SSRS reported by the participants’ mothers) at exactly the same ages, thus controlling for age differences in the design of the study. Among females, but not males, they found that pubertal status at baseline was negatively associated with initial self‐control in socioemotional settings (e.g., responding to teasing, peer pressure, and controlling temper) adjusting for sex, ethnicity, and baseline SES. Despite this, there were no longitudinal associations between pubertal status and self‐control.

Ng‐Knight et al. (2016) collected data on pubertal status (using the PDS) and hot self‐rated self‐control (using the BSCS, which measures the ability to resist temptations, control bad habits and avoid doing things they might later lead to regret) in a longitudinal study with three waves of data collection, each of which 6 months apart. The authors built a large structural equation model including parenting style measures, pubertal status, and hot self‐control, adjusting for sex, age, parental education, and the score for total difficulties from the Strengths and Difficulties Questionnaire (which combines the items for emotional symptoms, conduct problems, hyperactivity/inattention, and peer relationship problems). The results showed that more advanced pubertal status at baseline was associated with worse self‐control at baseline, similarly to Chaku and Hoyt (2019), but unlike the latter's female‐only association, Ng‐Knight et al. observed the effect in both sexes.

Warren and Brooks‐Gunn (1989) reported a similar finding but again only in females. While adjusting for age, the authors found a quadratic (but not a linear) association of Tanner stages and blood concentrations of different sex hormones with scores on the Impulse Control subscale of the Self‐image Questionnaire for Young Adolescents, a questionnaire that assesses hot SR/EF in the form of the capacity to control impulses under emotionally arousing conditions. Specifically, there was a decreased trend in impulse control during early puberty, followed by an improvement from mid‐ to late puberty.

Koch et al. (2020) did not show the same hot SR/EF difficulties in early puberty but, instead, found that self‐reported failures in regulating rumination increases throughout puberty (assessed using the PDS) in a sample of female adolescents. The same results were found by Mendle et al. (2020), who seems to have used largely the same sample as Koch et al. (2020). Mendle et al. (2020) also reported no significant effects of pubertal status (based on the PDS) on impulsivity when in a state of negative affect (negative urgency), assessed with the UPPS‐P scale (again, only females were assessed).

Vannucci et al. (2014) also failed to find significant effects of pubertal status (based on Tanner staging) on a measure of self‐reported loss‐of‐control eating in either male or female adolescents, and the single study that assessed social cognition (theory of mind on a story comprehension task) found no pubertal effects either (Vetter et al. 2013).

- Self‐regulation/executive function in socioemotional–neutral contexts (cool)

Three studies used this type of measure. No relation of pubertal status adjusted for age was found in scores in questionnaires that assessed cool controlled/effortful SR/EF, not associated with socioemotional context (e.g., difficulty sitting still, concentrating, shifting among activities, and persevering). This includes a comparison of a parent‐assessed Attention Subscale of the Child‐Behavioral Check List (CBCL) with PDS scores (Chaku and Hoyt 2019), and of the 5‐item Attention Scale from the Youth Self‐Report Questionnaire with blood levels of dehydroepiandrosterone (DHEA; Graber et al. 2006). There was one exception: the dissertation by Ellis (2002) reports small negative partial correlations (adjusting for age) between pubertal status in females (measured with the PDS), but not males, and overall self‐ and parent‐reported activation control, attention, and inhibition subscores of the Effortful Control subscale of the EATQ‐R.

- Classic laboratory tasks associated with cool self‐regulation/executive function abilities (e.g., executive functions, working memory, attention)

The remaining 11 selected studies assessed cool SR/EF abilities that can be categorized under the rubric of cool EFs using laboratory tasks that do not involve socioemotional contexts or arousing stimuli. Some of these abilities were assessed in only one or two studies, none of which were found to relate to pubertal status adjusted for age. This was the case for abilities such as verbal fluency, assessed in two datasets: Herlitz et al. (2013)/Lovén (2012), Waber et al. (1985). It was also the case for two of the measures used in Sullivan et al. (2016), from the University of Pennsylvania Web‐Based Computerized Neurocognitive Battery–WebCNP, including a composite of tasks involving sustained attention (including various measures of a continuous performance task) and another composite of abstraction (combining Conditional Exclusion Test, Matrix Analysis Test, and the Logical Reasoning Test).

Differently, there were two executive domains that were tested in more than a couple of studies, albeit with vastly different tasks/scores: inhibition of automatic responses, and visuospatial working memory abilities, the former having shown consistent lack of pubertal status effects corrected for age, while for the latter there were mixed results across puberty adjusting for age, as detailed next.

- Inhibition of automatic responses

Seven studies investigated inhibition measures. No pubertal effects adjusted for age were associated with performance on any of the tasks used. This includes associations of: the Multisource Interference task with PDS scores in Deater‐Deckard et al. (2019); the Antisaccade task with Tanner staging, the PDS, and with an average of PDS scores and Tanner stating scores in Ordaz et al. (2018), Ojha et al. (2022), and Ravindranath et al. (2022), respectively; the Go/No‐go task with PDS scores and salivary concentrations of estradiol, progesterone, DHEA, and testosterone in Gorday and Meyer (2018); and the Stroop Color‐Word Interference with Tanner staging in Waber et al. (1985).

The only exception to the lack of PDS scores, corrected for age effects, on executive functioning was the study by Castagna and Crowley (2021), who analyzed the parameters of two different decision models fitted to data from the inhibition Flanker Task and found a very small, negative effect of the interaction between sex and pubertal status on behavioral parameters representing the amount of information that is considered for a decision to be made (drift diffusion model). A follow‐up analysis of these interactions showed a negative relationship between pubertal status and inhibitory control.

- Visuospatial working memory abilities

Five studies investigated spatial working memory. Age‐adjusted pubertal effects were mixed, with positive effects (Davison and Susman 2001; Herlitz et al. 2013/Lovén 2012; part of the results from Kovács et al. 2022), as well as negative effect (Waber et al. 1985), and two reports of no effects (Sullivan et al. 2016; part of the results from Kovács et al. 2022).

Herlitz et al. (2013)/Lovén (2012) found a positive partial correlation, adjusting for age, between scores in a mental rotation task and estradiol concentrations in males but not in females, with more sexually mature males having better performance.

Similar results were reported by Davison and Susman (2001), who carried out cross‐sectional analyses using data from three waves of a longitudinal study (each 6 months apart). They looked at the correlation between hormonal levels and different measures of spatial abilities involving working memory (block design and the primary metal abilities test), analyzing data for males and females separately. There was a mix of positive and null results. Cross‐sectional analyses showed that in some waves there were positive correlations between mental rotation and testosterone for female participants. For male participants, there were positive correlations between testosterone levels and mental rotation scores, and between both testosterone and estradiol levels and scores in the block design task. The study also reported a longitudinal linear association between testosterone levels and mental rotation/block design for males, but not females.

In contrast, Waber et al. (1985) reported a significant age‐adjusted *negative* correlation between both coding and block design scores and Tanner stages in female participants, but found no association in males. However, this effect could only be found when separately analyzing data for participants from one of the two study towns, so should be interpreted with caution.

Sullivan et al. (2016) reported no association of PDS scores in either sex with a composite speed and accuracy score of working memory updating tasks (created from a combination of various measures of a visual N‐back task). There are limited details about these results in the paper.

Finally, Kovács et al. (2022) reported mixed results in a sample of female adolescents using two different sets of tasks related to working memory and visuospatial reasoning, both taken from the Wechsler Intelligence Scale for Children IV (Wechsler 2003). One set of tasks included the Block Design, Visual Puzzles, and Matrix Reasoning, and their scores were used to compose a Perceptual Reasoning Index. The partial correlation, adjusting for chronological age, between this index and physical maturation (measured by bone ultrasound) was not statistically significant. The second set of tasks included the Digit Span and Letter‐Number Sequencing, used to compose a Working Memory Index. This index showed a small, positive, statistically significant correlation with physical maturation as measured by bone ultrasound.

#### 3.3.1 A Glance at the Whole Picture—The Association of Pubertal Status and Self‐Regulation/Executive Function

The results reviewed in the previous sections highlight the significant heterogeneity of findings reported in this literature. However, a look at the information listed in Table 2, and a careful evaluation of the study details listed in Table S2, shows that results are even more heterogeneous than they seem from the descriptions provided above, because various details regarding the analysis of experimental data in each study were omitted for the sake of creating a readable summary.

It should be noted that some level of heterogeneity in the studies’ methods and results would be expected at the outset given the broadness of SR/EF as a construct (not to mention variations in study design and in pubertal measures). However, even after categorizing studies based on the tested SR/EF measures, considerable heterogeneity remained. In fact, even among studies claiming to assess the same ability or process, there was still large variability in methods and results, and often with too few studies per category to find reliable patterns in these variations.

It is thus no surprise that the results were rife with inconsistencies regarding the existence and direction of pubertal effects, as detailed in the Discussion. In most cases in which significant associations were found between age‐corrected pubertal status and a measure of some SR/EF ability, there were corresponding studies that assessed the same or similar SR/EF construct and that did not find statistically significant associations, or even found associations in the opposite direction.

There were only two domains in which more than a couple of studies assessed comparable outcome and showed reasonably consistent findings (though even here consistency was not perfect). The first was a lack of pubertal effects controlled for age in studies measuring inhibition in/with nonsocioemotional (cool) contexts/content (Waber et al. 1985; Gorday and Meyer 2018; Ordaz et al. 2018; Deater‐Deckard et al. 2019), with Castagna and Crowley (2021) being the exception.

A second apparently consistent finding was that more advanced pubertal indicators were related to more hot SR/EF difficulties assessed using questionnaires, an effect that was consistently present only in females in early‐puberty (Warren and Brooks‐Gunn 1989; Ellis 2002; Ng‐Knight et al. 2016; Chaku and Hoyt 2019; Koch et al. 2020/Mendle et al. 2020). This case was not perfectly consistent either, because Vannucci et al. (2014) found no effects of pubertal status on a measure of self‐reported loss‐of‐control eating using a sample that included both male and female 8–17‐year‐olds. Additionally, Mendle et al. (2020) reported no significant effects of pubertal status on impulsivity when in states of negative affect (negative urgency), while rumination (i.e., failure to control persistent negative thoughts) was found to increase progressively throughout puberty in females (Koch et al. 2020/Mendle et al. 2020).

### 3.4 Critical Evaluation of Methodological Issues in the Literature

We found a set of methodological issues that undermine our confidence on the internal and external validity of the findings reviewed here.

As discussed in the Methods section (see subsection “Criteria for study evaluation”), we focused our analysis on a set of aspects we judged as being critical for a more nuanced interpretation of the results in the literature. These include the study design, the characteristics of the participants, the instruments used to assess SR/EF abilities and pubertal status, and different issues related to the adequacy of the statistical analyses employed. In what follows, we discuss our analysis and its implications. We argue that the combined impact of all these issues is that, given the characteristics of the studies that have been carried out so far, it is as yet impossible to draw firm conclusions about whether and to what extent the general construct of SR/EF changes across pubertal maturation per se, independently of possible SR/EF changes that are expected to occur as adolescents grow older and gain experience.

#### 3.4.1 Study Design

One aspect is common to all of the reviewed studies is that they were observational studies—which, given our research question, is the only type of study possible. Moreover, the majority of the studies reviewed here were cross‐sectional investigations involving participants of different ages, or longitudinal studies with participants mostly of different ages at baseline. Because of this, and despite the complexities of the literature reviewed here, the lack of large, reliable and significant effects of pubertal status is hardly surprising.

We mentioned before that results that do not adjust for age effects are uninterpretable in terms of our research question. However, because of the strong correlation between age and pubertal status, the required statistical adjustments for participants’ age can artificially reduce pubertal effects. The shared variance of pubertal and age effects of the cognitive outcome is likely to be removed from the final effect when this type of statistical adjustment is used. Therefore, this approach avoids mistaken claims about the existence of pubertal effects, but increases the risk of missing real effects in observational studies.

In this scenario, the ideal solution would be to conduct longitudinal studies where participants are evaluated at the same ages, allowing the control of the effects of age without artificially reducing putative pubertal effects. That way it would be possible, in theory, to investigate to what extent pubertal development, as opposed to experience, learning, and nonpubertally induced physiological brain changes, influences cognitive/behavioral development.

Only one study in our sample, Chaku and Hoyt (2019), employed such a design. This study included around 1000 adolescents and assessed Tanner stages and parent‐reported questionnaires of attention and SR/EF yearly over a 7‐year period. However, data on SR/EF and attention were not collected in several study waves, including those encompassing the ages when most of pubertal development takes place. This significantly limited how informative this study could be to our review question, and may explain the lack of longitudinal pubertal effects.

#### 3.4.2 Sample Characteristics and External Validity

While we noted the heterogeneity of the studies included in this review, they were not heterogeneous in all aspects. Sample demographics were mostly similar among the majority of the reviewed studies. Overall (although with exceptions), the samples studied in this literature conformed to the general pattern that has been nicknamed W.E.I.R.D, meaning that participants were from western, educated, industrialized, rich, and democratic societies (Henrich et al. 2010).

The countries represented in this literature are far from homogeneous. The United States, the most represented country in this literature, has individuals of different ethnicities and from widely variable SES backgrounds, as can be seen in their most recent census (see https://www.census.gov/newsroom/press‐releases/2022/acs‐5‐year‐estimates.html and links therein). However, not even this within‐country diversity was fully represented in the samples. Based on reported demographic data—that is, from studies that actually reported such data—participants were mostly White and from middle‐ to upper‐class families.

There have been calls for increasing diversity in the study of adolescent development, because it is known that several factors related to ethnicity, culture, and SES can influence different aspects of adolescents physiological and psychological development (Herlitz et al. 2013; Steinberg et al. 2018; Blum and Boyden 2018; Mendle et al. 2019; Worthman et al. 2019; McLean and Riggs 2022). We stress, however, that this issue is not exclusive of the literature reviewed here, as researchers of different subdisciplines in behavioral analyses have become aware in recent years (Henrich et al. 2010, and associated commentaries; Hartmann et al. 2013; Fernández and Abe 2018; Amir and McAuliffe 2020; and associated commentaries). The lack of diversity in study participants is a widespread problem whose significance is still to be fully determined. One thing is for sure: this lack of diversity threatens the studies’ external validity. We simply cannot know how generalizable most of the reported results are, so interpreting them as results pertaining to “adolescents in general” creates a risk of bias.

#### 3.4.3 Issues With Cognitive/Behavioral Measures

As noted earlier, there was great variability among studies in the instruments employed to evaluate SR/EF. The literature is full of terminological disputes and divergent theoretical frameworks that can influence the choice of measures and the interpretation of what they assess, leading to the identified variability. It is unlikely that reliable conclusions can be draw by directly comparing the results of, say, a study using the attention subscale of the CBCL with the results of another study that tested attention using the Continuous Performance Test. While both measures may assess the same general‐domain underlying construct—“attention”—each task/questionnaire also captures other abilities, and these differ from one instrument to the other. This is a serious issue because elevated measurement error threatens the validity of study results in different ways, depending on the characteristics of the data and type of analyses (Carlson and Herdman 2012; Westfall and Yarkoni 2016; Loken and Gelman 2017). Of note, these issues are neither new in the study of SR/EF (e.g., Duckworth and Kern 2011; Fernández‐Marcos et al. 2018; Dias et al. 2024) or unique to this field (Meyer et al. 2001; Flake and Fried 2020).

One strategy to deal with measurement error and convergent/construct validity issues is the use of latent variable approaches under structural equation models (e.g., Royall et al. 2002; Westfall and Yarkoni, 201; Friedman and Miyake 2017; Karr et al. 2018). This method uses multiple tasks that are believed to measure the same construct and their shared variance (latent trait) is used as a dependent variable. The variance in performance that is unique to each variable is partialled out so that these latent variables are likely less impure and also freer of measurement error. However, the few selected studies that used more than one SR/EF measure aimed to assess distinct abilities with each measure, so latent traits could not be determined. Moreover, this type of analyses requires large samples, which were not used in the majority of analyzed studies.

#### 3.4.4 Issues With Pubertal Measures

The PDS was the most commonly used method to assess pubertal status in the studies reviewed here. The reasons for this prevalence are not hard to guess. The PDS is easy to apply, accessible, more affordable for researchers and less embarrassing for youngsters than other instruments. However, the PDS also has significant limitations, including the fact that it does not measure pubertal status per se—rather, it measures “perceived pubertal status” (Cheng et al. 2020). Accordingly, there are systematic discrepancies between PDS ratings and ratings from physical examination by clinicians, with less developed adolescents overestimating their development and more developed adolescents underestimating theirs (e.g., Shirtcliff et al. 2009).

In turn, Tanner staging based on physical examination by an experienced physician or nurse is regarded as the gold standard for assessing pubertal status (Dorn et al. 2006; Dorn and Biro 2011; Mendle et al. 2019; Walker et al. 2020) because it is believed to be more objective and reliable than self‐reported measures; however, it also has limitations. Tanner stages are based on a limited set of physical characteristics and rely on norms established several decades ago on a sample with limited diversity. Furthermore, it is not clear how objective and reliable this method is, as there can be substantial inter‐rater variability (Dorn et al. 2006; Dorn and Biro 2011).

Moreover, while Tanner stages are based on definite characteristics, it is possible—and we would say even probable—that clinicians’ rating of particular characteristics is influenced by all developmental signs that are perceptible during physical examination, not just those included in the Tanner method, as pointed out long ago by Morris and Udry (1980). Also, it requires having a clinician or trained nurses to do the evaluations in private, which substantially increases study costs.

In sum, both the PDS and Tanner stages have advantages and disadvantages, and both are indirect measures of puberty's unfolding—measuring the external signs of the underlying physiological processes that compose puberty (França et al. 2022). Because of this, the level of information they can deliver is limited by different factors, such as self‐report bias, observer error, and contextual confounders (Worthman et al. 2019).

Limitations also apply to measurement of hormone levels. At a first glance, these measures may be seen as a more “direct” and objective measure of pubertal developmental status because they reflect the immediate products of the neuroendocrine events that compose puberty. This seems especially true considering that the effects of pubertal status on cognitive abilities are likely caused by hormonal actions in the brain. Hormonal measures are thus proposed by some to be more informative than physical measures.

While there are strong correlations between hormones and physical measures of puberty (e.g., *r* values ranging from 0.6 to 0.7 in Shirtcliff et al. 2009), there is still a significant amount of variation in hormone concentrations that is not captured by the physical measures. In fact, pubertal hormonal levels cannot be clearly matched to stages of physical development (Dorn et al. 2006; Dorn and Biro 2011). This divergence between physical measures and hormone levels may have multiple causes—some related to the physiology of hormones, others related to the way they are measured.

Furthermore, while hormone measures are objective, there are many opportunities for variability and measurement error to creep in. Hormones can be measured from different samples (e.g., saliva, blood, hair) which may yield related results but are unlikely to produce perfectly equivalent ones. There are also different analytical procedures that can be used to measure the hormones, such as ELISA and radioimmunoassays, which have different sensitivities. Each of these approaches can produce heterogeneous results depending on the precise protocol, the antibody used, and so forth. Moreover, the time of day in which samples are collected and/or the way they are handled and stored before analyses may affect the results.

As for the physiology of the hormones, the determinants of hormonal effects include many other factors in addition to their circulating levels. These include the concentrations of hormone‐binding proteins, and the distribution, density, and particular isoform/genetic variant of hormone receptors in the different target tissues (Dorn et al. 2006; Ponzi et al. 2020). Some of these factors can vary both between individuals as well as within individuals over time—and with them the effects of hormone exposure (Auyeung et al. 2013; Schulz et al. 2009; Ponzi et al. 2020). These factors may contribute to the less‐than‐perfect correlations between hormones and PDS or Tanner, and to variations in the synchronicity of different aspects of physical changes during puberty. Importantly, these factors may also lead to significant individual variability in the association between hormonal levels and brain development.

Our sample of studies also included a single paper assessing pubertal status through bone age as measured by ultrasound parameters (Kovács et al. 2022). Like the PDS and the Tanner staging, this method is based on pubertal effects on specific tissues. However, like hormonal assays, it has the advantage of providing an objective measure. Nonetheless, the issues noted so far also apply here. First, like other physical measures, the correlation between bone age and gonadal hormones will depend on myriad factors that can affect the relevant tissue's sensitivity to these hormones. In the case of bone development, it is well‐known that other hormones, such as growth hormone, IGF‐1, thyroid hormone, and calcium‐regulating hormones play an important role and can significantly influence the process of bone development (Mallorie and Shine 2022). Second, similar to the case of hormonal measures, variations between measurement methods may have important effects on the observed results. It is unclear to what degree the results of pubertal effects based on bone age would vary depending on the methods used for measuring it. Commonly used methods of ultrasound and X‐ray imaging have each known advantages and limitations (De Sanctis et al. 2014), and while reported correlations between them are strong (*R* values ranging from 0.57 to 0.67 in Moris et al. 1995), they are far from perfect.

Despite their limitations, the fact is that PDS, Tanner staging, hormonal measures, and bone age are among the best measures of pubertal status we have right now. But they measure different things, and thus provide different information. Much like the notion of “development” itself, puberty is a concept we use to designate a complex set of interrelated processes, and can only be observed indirectly through these measures, so could be best characterized by the shared variance (latent variable) of many pubertal measures, because our current individual instruments offer a rather noisy picture of this construct (for a more detailed discussion of these issues, see França et al. 2022). Because we cannot know all the factors that determine the effects of pubertal hormones on the brains of each individual, and because the effects of pubertal hormones vary from tissue to tissue, an estimation of pubertal status based on multiple indicators is probably the most reliable and robust measure we can obtain. Latent pubertal measures could even be improved over time in the literature by adding more measures as new methods and biomarkers are developed. The incorporation of latent constructs in studies involving puberty has been called for before (Dorn and Biro 2011) and we have seen recent steps in this direction (Byrne et al. 2019; Herting et al. 2021), although there is still a long way to go before this strategy becomes widespread. Hence, future studies should strive to obtain multiple measures of puberty and associate the effects of a latent variable obtained from them with SR/EF abilities.

#### 3.4.5 Statistical Issues

Statistics is involved in most of the issues already discussed above. It is relevant to the problems of measure (un)reliability, and also plays a key role in the solution of this problem. Furthermore, the whole issue surrounding the confounding effects of age is essentially a statistical one. However, there are two additional statistical difficulties we have not yet discussed. The first one is the significant variability in the reporting of statistical results and in the characteristics of statistical models employed; the second pertains to linearity—or lack thereof.

A significant source of difficulty when interpreting and comparing results was the variation in the statistical approaches used in each study, including significant diversity in the number and identity of control variables included in the statistical models. Besides adjusting their analysis for age, which was a requirement for inclusion in the review, most studies adjusted their analyses for some known confounders together with the variables of interest to each study. Variables added to statistical models included sex, SES, ethnicity, intelligence, among others. However, no study adjusted for all those variables, some adjusted for no variables other than age, and others included covariates in their models that were of no interest to our review.

There was also great variability in the precise statistics reported in each study (e.g., correlation or partial correlation coefficients, coefficients of determination, standardized or unstandardized regression coefficients, all of which mostly lacking in, or varying in the reported confidence intervals). Also, when significant associations were found, they were mostly of small magnitude (although there were exceptions). This makes it difficult to evaluate, let alone compare, the magnitude and precision of effects from different studies.

As for the second issue, most of the statistical models used in the reviewed studies were linear models (which exception of part of the analyses in Warren and Brooks‐Gunn [1989], Davison and Susman [2001], and Ordaz et al. [Ordaz et al. 2018])—as is standard for most experimental fields in psychology and biology. These models may not be the best ones for dealing with biopsychological development during adolescence because most of the developmental processes during this period are nonlinear: pubertal development is not linear (e.g., Marceau et al. 2011), brain development during adolescence is not linear (e.g., Foulkes and Blakemore 2018; Gracia‐Tabuenca et al. 2021), behavioral changes during adolescence are not linear (e.g., Steinberg et al. 2018). In fact, when the subject is adolescent development, it seems that nothing is linear except our models.

The issue of nonlinearity, however, may be more serious when studying adolescents from a wider age range. For studies that involve participants of a short age range, linear models may be a good approximation of the developmental effects/trajectories. Nonetheless, to fully understand the developmental processes occurring during adolescence, statistical models that better capture the pubertal transition as a whole are needed. Accordingly, there have been calls for the development of nonlinear approaches in the literature, and nonlinear models are starting to be explored with promising results, but here too much still remains to be done (Susman et al. 2019; Worthman et al. 2019).

## 4 Discussion

Our primary goal in this systematic review was to establish the state of the evidence regarding the association of pubertal status with behavioral SR/EF performance in adolescents. The overall picture that emerges from our review, however, is that of a literature rich in data but poor in reliable answers. Importantly, because of the confounding effects of age, which is associated with both pubertal status and SR/EF, we looked for studies that controlled or adjusted for age effects. This is a necessary condition to interpret results in terms of effects of the stage of pubertal development on SR/EF, irrespective of when puberty begins (timing) or how long it takes to end (tempo).

However, for varied reasons, most of the literature did not meet this criterion. In fact, we found a total of 125 studies reporting statistical results on the relationship between pubertal status and SR/EF, but 96 of these were not interpretable in the context of our research question, and almost all interpretation issues were caused by either not controlling/adjusting for age effects or doing so in an insufficient/inappropriate way to provide information on pubertal status effects—either because the adjustment was not sufficiently fine‐grained or because it fundamentally altered the pubertal variables, as detailed below.

This meant that we were left with 29 documents, reporting a total of 28 studies from 27 datasets, which adjusted or controlled for age effects in their analyses of the relationship between pubertal status and SR/EF. However, the picture formed by synthesizing these studies is difficult to interpret. The studies reviewed here employed a limited set of the currently available instruments to assess hot and cool SR/EF, covering an equally limited set of constructs and abilities related to SR/EF.

There was also considerable heterogeneity among studies in terms of the SR/EF constructs/measures under investigation, and the tests/questionnaires that were used. Even studies that claimed to assess the same construct often used different instruments or different scores that were difficult to compare. This created considerable interpretation difficulties, especially when comparing studies with conflicting results, because it could not be clearly established whether these results truly disagreed or merely reflected the involvement of different types of SR/EF. The heterogeneity in this literature was further increased by the use of varied pubertal indicators that are not directly comparable, either because of the lack of a clear match among different physical changes that index pubertal development, or due to a lack of a match between these physical markers and sex hormone concentrations (Dorn and Biro 2011; Mendle 2014; Mendle et al. 2019; França et al. 2022).

The reported results included a mix of nonsignificant and significant findings. This, in itself, would be natural given the random variation intrinsic to any sampling, the fact that the reviewed literature typically used relatively small sample sizes, and that studies often made use of complex statistical models. More troubling is the presence of results with effects in opposite directions, even when studies claimed to assess the same constructs, and used similar instruments.

Overall, there were few clear patterns in the distribution of positive, negative, and null result among studies when considering the general type of the abilities assessed (e.g., hot vs. cool SR/EF), the instruments used (tasks vs. questionnaires), or the particular construct assessed (e.g., inhibition, working memory, impulsivity, etc.).

### 4.1 Does the Evidence Support the Predictions of Cognitive Developmental Models?

With a patchy coverage of SR/EF abilities, and high heterogeneity in measures, scales, and analyses, a lack of clear patterns should be no surprise. This general inconsistency was observed for performance in measures that assess decision‐making under risk/uncertainty and sure wins, generally regarded as hot skills, as well as for (cool) visuospatial working memory. Despite this, there were two apparently consistent sets of results that could inspire tentative conclusions considering the most cited models of cognitive development during adolescence.

The first is the almost complete lack of reported effects in studies involving questionnaires of cool SR/EF in real‐life scenarios (Graber et al. 2006; Chaku and Hoyt 2019; except for a very small effect in females only: Ellis 2002) and laboratory tasks that involve SR/EF abilities like access to long‐term memory (verbal fluency: Waber et al. 1985; Herlitz et al. 2013/Lovén 2012), working memory updating and attention (Sullivan et al. 2016; Kovács et al. 2022) and, more markedly, inhibition, which was tested in many studies, albeit mostly using different tasks (Waber et al. 1985; Gorday and Meyer 2018; Ordaz et al. 2018; Deater‐Deckard et al. 2019; Ojha et al. 2022; Ravindranath et al. 2022; with the exception of a small female‐specific effect in Castagna and Crowley 2021). The exception to this general lack of pubertal effects on cool SR/EF occurred for visuospatial working memory tasks, which showed mixed results: while Davison and Susman (2001), Herlitz et al. (2013)/Lovén (2012), and Kovács et al. (2022) found small to medium positive effects in at least part of their analyses, Waber et al. (1985) and Sullivan et al. (2016) found no consistent pubertal effects.

The second pattern in the results was the recurrent finding of negative pubertal effects on day‐to‐day hot SR/EF skills assessed with questionnaires, including failures in self‐control (Ng‐Knight et al. 2016; Chaku and Hoyt 2019), impulse control (Warren and Brooks‐Gunn 1989; Ellis 2002), and emotional regulation (Koch et al. 2020/Mendle et al. 2020).

Together, these two apparent patterns (lack of pubertal effect on cool SR/EF abilities and negative pubertal effect on hot SR/EF in real‐life settings), seem in line with prevailing imbalance models of cognitive development across adolescence, which propose that only hot abilities are associated with puberty (Shulman et al. 2016). Regarding cool SR/EF, the imbalance models posit that there is a gradual/linear pubertal‐independent improvement throughout adolescence due to the protracted development of the prefrontal cortex (for reviews, see Ernst et al. 2006; Ernst 2014; Casey 2015; Shulman et al. 2016). Thus, when age is corrected for, no pubertally associated change in cool SR/EF should be found, as observed in the majority of studies here.

The picture changes, however, when considering the prediction of the imbalance models in terms of the findings for hot SR/EF effects. These models propose that the approach/reward‐driven system involving mainly striatal regions, develops along a nonlinear trajectory dependent on pubertal status (Casey 2015; Shulman et al. 2016). More complex imbalance models, such as the triadic model (Ernst et al. 2006; Ernst 2014), go further by proposing a third, avoidance‐related system centered on the amygdala, that also develops nonlinearly under puberty's influence. In both cases, the main prediction from these proposed developmental trends is a peak in reward sensitivity and/or increased impulsiveness under conditions involving social or emotional responses in the midteen years (Ernst 2014; Casey 2015; Shulman et al. 2016). This peak would coincide with the end of puberty for most individuals (Marceau et al. 2011).

At first glance, the predictions above seem compatible not only with findings of an increase in SR/EF failures from questionnaires assessing hot SR/EF in real life (Warren and Brooks‐Gunn 1989; Ellis 2002; Ng‐Knight et al. 2016; Chaku and Hoyt 2019; Koch et al. 2020/Mendle et al. 2020), but also with the findings of four studies that showed higher pubertal‐related choice impulsivity in tasks that assess hot decision‐making involving rewards (Kretsch and Harden 2014; Cardoos et al. 2017; Laube et al. 2020, 2017). However, this apparent agreement does not hold under further scrutiny.

First, given that the reports using questionnaires about daily life failures in hot SR/EF were in agreement only regarding early puberty effects, and exclusively in females (Warren and Brooks‐Gunn 1989; Ellis 2002; Ng‐Knight et al. 2016; Chaku and Hoyt 2019; Koch et al. 2020/Mendle et al. 2020), it may very well be that they do not relate specifically to SR/EF capacities. This observed effect could be related to difficulties/inexperience in dealing with the beginning of mood fluctuations following the first menstrual cycles, a symptom that can affect up to 80% of females (Itriyeva 2022). Second, the lack of pubertal effects on negative urgency (Mendle et al. 2020), loss‐of‐control eating (Vannucci et al. 2014), and social cognition difficulties (Vetter et al. 2013), do not square well with the models’ predictions, as negative effects on hot SR/EF would be expected in these studies. Third, most results that indicated worse hot SR/EF in real life were observed in early puberty, which usually *precedes* midadolescence, contrary to the models’ predictions in terms of the effect's timing.

There were also many inconsistencies among studies which assessed hot SR/EF questionnaires covering aspects relevant to the dual‐system models. For example, some studies reported nonlinear trends (Warren and Brooks‐Gunn 1989), others reported impairments throughout the whole pubertal transition (Koch et al. 2020/Mendle et al. 2020), some only in early puberty (Ng‐Knight et al. 2016; Chaku and Hoyt 2019), and still others reported no effects at all (Vetter et al. 2013; Vannucci et al. 2014; Mendle et al. 2020).

Another point of disagreement between the literature and model predictions can be seen in the studies using hot decision‐making laboratory tasks. While four of those studies showed negative pubertal effects, as predicted by imbalance models (Cardoos et al. 2017; Kretsch and Harden 2014; Laube et al. 2020, 2017), two studies showed the opposite effect (Steinberg et al. 2008; Icenogle et al. 2019), and three others found no effects of pubertal status (Olson et al. 2009; Sullivan et al. 2016; Lee and Rasmussen 2022).

Furthermore, we failed to see a differential pattern in results involving hot skills under risks/uncertainty (e.g., gambling, risky driving) versus safe options with sure wins (e.g., delay discounting). These two classes of tests should be differently affected across adolescence according to current models (Defoe et al. 2015; Romer et al. 2017). Specifically, greater impulsivity is supposed to be more prevalent under uncertainty and to be related to puberty, whereas better choices involving sure‐win options should increase with age and experience. Specifically, a higher risk‐taking profile (in tasks such as the Iowa Gambling Task, the Airport Auction Task, and simulated driving in the Stoplight Game) following pubertal status was found in two of four studies (Kretsch and Harden 2014; Cardoos et al. 2017), the other two of which found that responses became *less* risky as adolescents became more pubertally mature (Steinberg et al. 2008; Icenogle et al. 2019). Regarding choosing between sure‐win options, two studies reported higher pubertal status‐related preference for immediate rewards in delay discounting tasks (Laube et al. 2020, 2017), but three other studies using the same type of paradigm found no effect (Olson et al. 2009; Sullivan et al. 2016; Lee and Rasmussen 2022).

Moreover, a different reading of the predictions of the imbalance models is that hyper‐responsive affective systems could lead adolescents to be more, and not less willing to wait longer for larger real rewards rather than preferring to gain less at shorter intervals (Scheres et al. 2014; see also Defoe et al. 2015). It is also noteworthy that none of the studies that used delay discounting tasks actually had adolescents wait to gain rewards, which can be aversive and lead to a preference for immediate choices. The five studies seem to have been entirely hypothetical, asking participants to imagine how long they would be willing to wait to gain virtual rewards. It is thus difficult to ascertain that these delay discounting tasks were really measuring heightened sensitivity to *real* rewards or aversion to having to really waiting for them. Regrettably, other types of choice/risk‐taking paradigms (reviewed by Defoe et al. 2015) were not tested in the selected studies in our review so we could not elaborate further on this matter.

Our results also failed to be informative regarding the predictions of another theoretical perspective on behavioral SR/EF in adolescence, namely, social engagement shifts across this period of life (e.g., Nelson et al. 2016). Only two reviewed studies investigated the effects of social manipulation/social cognition: Vetter et al. (2013; theory of mind) and Kretsch and Harden (2014; taking risks when watched by peers). Neither of them found pubertal effects related to performance. As a whole, the reviewed studies did not consistently confirm any predictions of developmental models, except that cool SR/EF measured with laboratory tasks is more consistent in not associating with pubertal status once age is accounted for.

### 4.2 Summary and Going Forward

When talking about future directions, we find ourselves in a situation that resembles the serenity prayer. There are difficulties we can resolve, and there are those we cannot—we must accept the former and try to solve the latter, but first we must know the difference. Thus, faced with the challenges of disentangling the cognitive and behavioral effects of pubertal status from other developmental factors that are not puberty‐related and that change as adolescents age (e.g., learning/gaining experience), the first question we may ask is, can we really do it? Apart from information that can be gathered from clinical cases of individuals with (neuro)endocrine pathologies, our main tool for investigating the effects of pubertal status on SR/EF in humans are observational studies. However, the deep interactions between the effects of growing older and going through puberty on the development of cognitive skills make it very difficult to disentangle their effects.

Some effects of pubertal status may well be nonseparable from those of experience at the level of behavior. For example, there is evidence that increases in estrogen concentrations during puberty can change the inhibitory tone in the frontal cortex of mice (Piekarski et al. 2017). Such changes would not only affect the neural representations in this region, but could also affect neuronal plasticity. In fact, increases in the activity of inhibitory interneurons are linked to the opening of sensitive periods in other brain regions at different stages of development (Hensch 2005; Takesian 2013). Thus, this effect of estrogen may not only lead to an “immediate” change in behavior (by changing the dynamics of neural representations), but also change the effects of experience over time through its effects on plasticity. This latter effect would involve an interplay between a pubertal effect and learning from experience.

There is also evidence that sex hormones influence various aspects of behavior, including mood and social cognition (Schulz et al. 2009; Auyeung et al. 2013; Schulz and Sisk 2016). This can alter how adolescents perceive different stimuli/situations (Nelson et al. 2016, 2014) and how others perceive and react to adolescents (Arnett and Twenge 2008), thus influencing the process through which individuals shape their environments over time, which then feeds back to influence subsequent development (Scarr and McCartney 1983).

This situation is further complicated by possible effects of other pubertal constructs, such as pubertal timing and tempo, be they absolute or relative to same age/sex peers. While discussing the vast literature on the psychological effects of these constructs is beyond the scope of the present review, it is worth noting that they may alter the effects of future pubertal status on cognitive and behavioral development. Maturing earlier or later than ones’ peers, or going through puberty fast versus displaying a more protracted trajectory, could have significant psychosocial effects, both directly (e.g., effects on self‐esteem, anxiety, and depression symptoms) as well as indirectly through the adolescent's social environment (e.g., affecting the way the adolescent is treated by parents and teachers). In fact, there is ample evidence for such effects (Mendle 2014), and all of them could impact cognitive and behavioral development. At present it is unclear what is the best approach to combine the effects of these different pubertal dimensions, especially since different pubertal constructs may affect different psychological dimensions, which then influence one another, creating a complex causal web.

More generally, discussed by Ernst (2014), adolescence is a time of life in which a complex balance between transient and trait factors lead to large interindividual differences in behavior, which may make it even harder to find a clear pattern of responses considering pubertal effects, especially when using small samples, as was the case of most reviewed studies. Transient factors include not only physical states such as pubertal status, but also mental states and mood, context (home, school, or nonsocial environments such as the laboratory), progressive reattribution of social values (Ernst 2014), as well as the opportunities to take risks in real life, the levels of risks involved in decision‐making, and the size of possible rewards (Defoe et al. 2015; Romer et al. 2017), all of which can interact in different ways. These changes are also influenced by trait factors such as genetics, personality, and past experiences (Ernst 2014).

Taken together, we believe these issues will require statistical models that are more informed by theory—a need already highlighted in the literature (e.g., Susman et al. 2019). Developing the theoretical insights to inform these models, however, will be a challenge on its own. Currently, the most popular models involving the development of SR/EF during adolescence are various dual systems and the triadic models (reviewed by Ernst et al. 2006; Ernst 2014; Casey 2015; Shulman et al. 2016). Less cited models highlight the effects of learning by accumulation of experience in decision‐making, which differs throughout adolescence depending on a host of factors such as risk probabilities (reviewed by Defoe et al. 2015; Romer et al. 2017).

While these models have many conceptual similarities, they vary in their emphasis on the role of different brain regions or networks, of learning by experience, and in their precise behavioral predictions, including their proposed trajectories of behavioral change. As discussed above, although some of the results seemed to agree with the general tenets of the imbalance models, overall there were many inconsistencies and findings that did not square with these models’ basic predictions.

We also failed to find consistent effects regarding decision‐making performance under risks and sure wins, resulting in a lack of evidence supporting models that underscore these variables as essential to describe adolescent behavior. Therefore, the literature reviewed here does not provide support for any of these models. In fact, evidence for the imbalance models—and especially to disambiguate between them—has been hard to come by as there are some important theoretical and methodological difficulties in testing them that have not been appropriately met (Pfeifer and Allen 2012, 2016; Meisel et al. 2019).

Currently, the triadic model (Ernst et al. 2006; Ernst 2014) seems to be more comprehensive in explaining pubertal effects because it also accounts for avoidance behavior in aversive situations by considering, in addition to the two systems of dual‐systems models, a third, emotion‐related neural system that involves the amygdala, hippocampus, and insula. Higher behavioral difficulties, associated with puberty, affecting the intensity and liability of emotional responses (possibly involving altered social salience) may relate to this third module. Teasing these models apart will require studies using sets of tasks that are sensitive to differences in frontal, striatal, and amygdala networks and that take social aspects and risk probability versus choosing between sure‐win options into account, preferably in the same individuals, with longitudinal designs, large samples, and a host of other factors discussed above.

Further development and refinement of rigorous and testable theories and models will require the integration of all the evidence available. This means combining insights from both human and animal studies, as the latter allow for the investigation of plausible mechanisms and how they may limit possible detectable effects at the behavioral level, helping to set realistic expectations for the results of future studies. We will also need to integrate information from investigations of pubertal processes involving different physiological systems and at different levels of organization, from the molecular to the behavioral level. In other words, we will need all the information we can get to appropriately constrain our models and provide a context for the interpretation of future results (Susman et al. 2019).

Considering all the issues raised in this review so far, we believe that, if we are to have a chance of disentangling the effects of pubertal status on the development of SR/EF, we will need: (1) more longitudinal studies; (2) studies with more diverse samples; (3) studies using multiple measures of pubertal development and theory‐oriented measures of SR/EF, with adequate statistical methods to deal with measurement error in individual instruments (e.g., latent variables); and (4) more studies with nonlinear, theory‐informed models. Of note, points 2, 3, and 4 are actually more urgent than point 1, because there is no use in conducting large longitudinal studies when there are still uncertainties about what to measure and how to do so.

Additionally, we should invest in the creation of public databases to allow full use to be made of the available data. Pubertal data can be analyzed in multiple ways, and thus the same data can be used to answer very different questions, as the diversity of questions investigated with data from the large ABCD study illustrates (Cheng et al. 2020). In fact, the data from most studies listed in Table S1, as well as data from studies with relevant measures but that did not report results for the associations between pubertal status and cognitive/behavioral outcomes of interest, could be used, at least in theory, to help answer our review question. Making data publicly available would allow the scientific community to make the most out of data—a moral imperative, given that most scientific studies receive public funding.

Finally, this move toward open datasets should include greater transparency in research reports. In fact, the most common methodological issues found in the studies reviewed here were limitations associated with incomplete reporting of the demographic characteristics of participants. Often, there was also insufficient information about cognitive and pubertal measures and statistical analyses and results, which created difficulties for interpreting the findings, and would certainly create difficulties for replicability and reproducibility. Additionally, data availability statements were only available for three studies (Chaku and Hoyt 2019; Kovács et al. 2022; Ravindranath et al. 2022), and no study mentioned a preregistered protocol.

The problem of transparency is particularly important when talking about measures such as tasks and questionnaires. For example, more often than not, full questionnaires are not made available with the research reports, with papers describing only a few sample items from the questionnaire. The full questionnaires can be proprietary tools, inaccessible to researchers with low resources, or be hidden behind a never‐ending trail of references, where one paper cites another as the source, which in turn cites another, and so on, with the original full questionnaire being difficult or even impossible to locate (this also applied to methods of analyses of hormonal concentrations). This issue is not particular to this literature, so greater use of open‐access pubertal and SR/EF measures and transparency would go a long way in allowing the development of a more inclusive and democratic field of research, as well as a deeper appreciation of the data that has accumulated over decades about cognition in adolescence.

### 4.3 Review Limitations

Disagreements about the definition of SR/EF and related concepts—which are rife in the literature—could have led to different criteria for including tasks and questionnaires in this review, as well as in the way we categorized them into hot and cool measures and so forth. In addition, our search for gray literature could have involved additional methods, like searching Google Scholar, thesis/dissertation repositories and Open Grey which, however, is being discontinued. We also did not attempt to obtain original datasets from all of the studies to reanalyze them for the purposes of our review question, which is beyond the scope of the present work and unlikely to have contributed to the discussion in light of the variety of measures and methods involved in the selected papers. We note, however, that the limitations identified in the literature reviewed here are widespread, and they significantly, and fundamentally limited how informative available data were in respect of our review question. Therefore, we do not believe any of the limitations above would significantly change the main conclusions of this review. An additional limitation was our focus on typically developing adolescents. Clinical groups may show different patterns of relationship between pubertal development and SR/EF abilities.

## 5 Conclusion

Our review revealed significant heterogeneity in the methods and results of the available studies about the relationship between pubertal development and SR/EF. There were only a few seemingly consistent findings when age is adjusted for, but which were of questionable significance: (1) a lack of pubertal effects on cool SR/EF, although very few studies used the same cognitive measures; and (2) a tendency to display difficulties in hot SR/EF when considering more ecological, day‐to‐day abilities, but exclusively around the onset of puberty in girls (with exceptions), which could be associated with pubertal changes unrelated to SR/EF itself (e.g., mood changes).

Although there is widespread conviction that pubertal status affects SR/EF, we found no conclusive evidence for this effect in typically developing humans. This is not to say that there is no evidence for the effects of pubertal status on SR/EF. Animal studies, for example, do suggest that pubertal development influences brain regions generally involved in these abilities, which nonetheless are inherently human. Notwithstanding, considering the available literature, it is not possible to confirm the existence and magnitude of purported specific effects of pubertal status on SR/EF in humans at the level of behavior due to the lack of studies in representative samples from different populations, especially longitudinal investigations, the use of few and/or distinct measures both of pubertal status and of cognitive abilities related to SR/EF in different studies, and the inappropriate methodological design for the purpose of disentangling the effects of pubertal development from age effects.

## Conflicts of Interest

The authors declare no conflicts of interest.

## Supporting information




**Supporting Table 1**: **S1** lists studies excluded for not addressing the review question; **Supporting Table 2**: provides further details about the studies included in the review.

## Data Availability

All relevant data is openly available in the porject's Open Science Framework page: https://doi.org/10.17605/OSF.IO/XFNUZ.

## Endnotes

1 Of note, the term “pubertal timing” is often used in the literature when referring both to the beginning of puberty or the onset of different pubertal milestones, and to timing or status compared to same age and same sex peers (e.g., Dorn and Biro 2011; Mendle 2014; Mendle et al. 2019), the later having potentially important social implications and being a construct of interest in its own right. We believe it is important to distinguish between these two uses. Therefore, we use “absolute pubertal timing” to refer to the beginning of puberty/age of onset of particular pubertal milestones, and “relative pubertal timing” as a measure of effects of the differences between same‐age, same‐sex peers in presenting these milestones.

2 We originally intended to search the OpenGrey database as well. However, preliminary searches on this database, conducted on May 2021 using the same search terms for other databases, returned only 14 results, none of them relevant for the review. Moreover, OpenGrey is being discontinued. Therefore, we opted to exclude this database from the review. We also considered, in the early phases of designing the review protocol, using preprint serves to search for unpublished studies, particularly the PsyArXiv and bioRxiv. However, at the time their search engines did not handle well complex search strings like the ones used in this review.
